# Optical control of a receptor-linked guanylyl cyclase using a photoswitchable peptidic hormone†
†Electronic supplementary information (ESI) available: Details on solid phase peptide synthesis and characterisation of all peptides can be found here, as well as experimental details on cGMP assays, aortic tensometry, islet treatment, statistics and a detailed description of modelling and simulations. See DOI: 10.1039/c6sc05044a



**DOI:** 10.1039/c6sc05044a

**Published:** 2017-04-19

**Authors:** Tom Podewin, Johannes Broichhagen, Christina Frost, Dieter Groneberg, Julia Ast, Helena Meyer-Berg, Nicholas H. F. Fine, Andreas Friebe, Martin Zacharias, David J. Hodson, Dirk Trauner, Anja Hoffmann-Röder

**Affiliations:** a Department of Chemistry and Center for Integrated Protein Science , LMU Munich , Butenandtstr. 5-13 , 81377 Munich , Germany . Email: anja.hoffmann-roeder@cup.lmu.de ; Email: dirk.trauner@cup.lmu.de; b Department of Physics , Technical University of Munich , James-Franck-Str. 1 , 85748 Garching , Germany; c Julius-Maximilian-University Würzburg , Institute of Physiology , Röntgenring 9 , 97070 Würzburg , Germany; d Institute of Metabolism and Systems Research (IMSR) and Centre of Membrane Proteins and Receptors (COMPARE) , University of Birmingham , Edgbaston , B15 2TT , UK; e Centre for Endocrinology , Diabetes and Metabolism , Birmingham Health Partners , Birmingham , B15 2TH , UK

## Abstract

The optical control over biological function with small photoswitchable molecules has gathered significant attention in the last decade. Herein, we describe the design and synthesis of a small library of photoswitchable peptidomimetics based upon human atrial natriuretic peptide (ANP), in which the photochromic amino acid [3-(3-aminomethyl)phenylazo]phenylacetic acid (AMPP) is incorporated into the peptide backbone. The endogeneous hormone ANP signals *via* the natriuretic peptide receptor A (NPR-A) through raising intracellular cGMP concentrations, and is involved in blood pressure regulation and sodium homeostasis, as well as lipid metabolism and pancreatic function. The *cis*- and *trans*-isomers of one of our peptidomimetics, termed TOP271, exhibit a four-fold difference in NPR-A mediated cGMP synthesis *in vitro*. Despite this seemingly small difference, TOP271 enables large, optically-induced conformational changes *ex vivo* and transforms the NPR-A into an endogenous photoswitch. Thus, application of TOP271 allows the reversible generation of cGMP using light and remote control can be afforded over vasoactivity in explanted murine aortic rings, as well as pancreatic beta cell function in islets of Langerhans. This study demonstrates the broad applicability of TOP271 to enzyme-dependent signalling processes, extends the toolbox of photoswitchable molecules to all classes of transmembrane receptors and utilizes photopharmacology to deduce receptor activation on a molecular level.

## Abstract 2

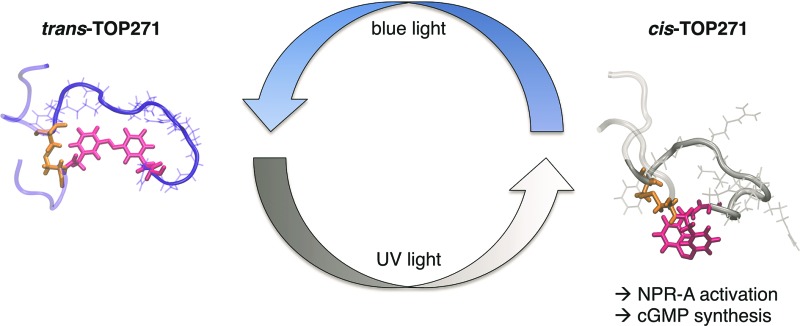
The photoswitchable peptidomimetic hormone TOP271 allows the precise optical control of cGMP generation *via* the receptor-linked enzyme NPR-A in explanted aortic rings and islets of Langerhans.

## Introduction

Controlling biological function with light has been achieved using two general approaches, *viz.* optogenetics^1^ and photopharmacology.^2,3^ While the first relies on the genetic introduction of light-responsive proteins, the latter describes the exogenous use of small photochromic molecules that interact with a specific target. The advantage of photopharmacology is the precise control of cell signalling through native receptors, without necessarily introducing foreign genes. While optogenetics has successfully targeted the receptor-linked enzyme (RLE) class,^4^ in particular receptor tyrosine kinases,^5,6^ photopharmacology has not kept pace. One reason is that RLE ligands are usually large peptides with few known small molecule activators, making it a challenge to find a suitable “azologable”^3^ pharmacophore. However, we and others recently reported the optical control of cell function with photoswitchable peptides,^7,8^ an approach that is highly applicable to RLEs.

**Fig. 1:**
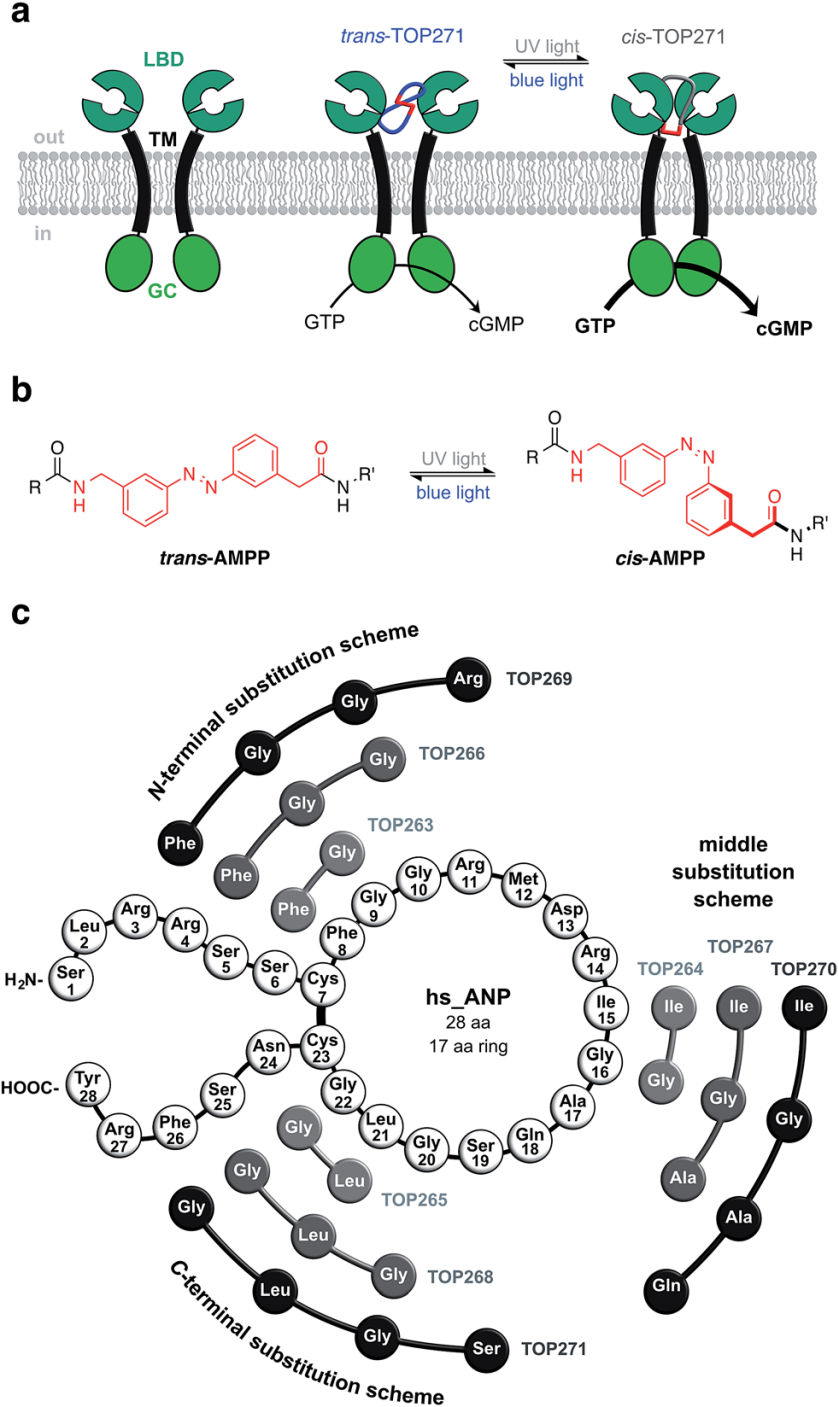
ANP receptor and AMPP. (a) The ANP receptor links photoswitchable peptide binding to activation of guanylyl cyclase, that is cGMP generation and subsequent vasodilation in its UV-light adapted *cis*-state. (b) The photoswitch AMPP can be toggled between its *trans*- and *cis*-isomer with blue (460 nm) and UV-light (365 nm), respectively. (c) Initial assessment of AzoANP peptides TOP263-271 potency towards cGMP synthesis focuses on TOP271, where four amino acids are replaced on the C-terminal side of the macrocycle.

Accordingly, we focused on the natriuretic peptide receptor A (NPR-A), with its endogenous agonist atrial natriuretic peptide (ANP), as a suitable target for RLE photocontrol (Fig. 1a). The physiological actions of ANP are widespread and range from blood pressure regulation and sodium homeostasis to effects on fat metabolism and pancreatic beta cell function/survival.^9–11^ ANP is mainly expressed and stored as inactive proANP in atrial cardiac myocytes, with lesser concentrations found in the ventricles and kidneys. Upon secretion, primarily controlled by mechanical stimulation following atrial wall stretching,^12,13^ proANP is rapidly cleaved by the cardiac serine protease corin to release the active 28 amino acid ANP.^14^ The active form comprises a central 17 amino acid macrocycle linked by a disulfide bridge between Cys7 and Cys23. Following ligand activation of NPR-A, the membrane-proximal regions of the monomeric receptor units undergo a global conformational change, triggering guanylyl cyclase activity. This leads to generation of cGMP, a major player in intracellular cell signalling.^12^

Dysregulated ANP secretion has been linked to different cardiovascular diseases, *i.e.* atrial fibrillation,^15^ hypertension^16,17^ and heart failure.^18,19^ Moreover, genetic variants in or close to the ANP gene (NPPA) locus, which lead to increased circulating levels of plasma ANP, were shown to lower blood pressure and the risk of hypertension in healthy individuals.^20,21^ Furthermore, individuals harbouring one copy of the G allele of rs5068 have lower likelihood of diabetes,^22^ and ANP has been shown to increase muscle insulin sensitivity,^23^ although whether insulin release itself is stimulated is more debated.^10,11,24^ Such fundamental and pleiotropic actions of ANP have made its receptors an important pharmacological target, resulting in recently introduced therapies for the treatment of cardiovascular diseases.^25,26^ Despite this, many facets of ANP function and action remain elusive. Thus, the development of novel tools for unravelling and controlling ANP/NPR-A-stimulated signalling processes would be a valuable asset.

To address this, we report the synthesis of a photochromic ligand based on human ANP that enables the photocontrol of RLE activity (Fig. 1a). The NPR-A was endowed with light-sensitivity by incorporation of the photoswitchable amino acid [3-(3-aminomethyl)phenylazo]phenylacetic acid (AMPP)^27,28^ into ANP, which along with related derivatives,^8,29–31^ has proven to be a valuable building block for photocontrol of peptide conformation and activity (Fig. 1b). One out of nine of our synthesised photochromic ANPs (AzoANPs), termed TOP271, allowed optical control to be exerted over NPR-A activity, intracellular cGMP levels, and downstream processes using UV and blue light.

## Results and discussion

Our initial design approach was based on the incorporation of AMPP into the peptidic backbone of ANP, to induce maximal structural changes upon photoisomerisation. Nine different photochromic AzoANP peptides (dubbed TOP263-271) were designed and synthesised to obtain a small library (Fig. 1c), whereby AMPP replaced two, three or four amino acids in ANP. These numbers were based on our experience with other peptides, in which AMPP displaced two amino acids and the fact that AMPP covers up to four amino acids in length.^8,28^ The substitutions, following a circular permutational fashion, were located either near the N- or C-terminus or facing the Cys7-Cys23 disulfide bridge in the native 17 amino acid cycle of ANP (see ESI† for details on synthesis and characterization).

For the incorporation of azobenzenes into cyclic peptides several approaches have been developed in recent years. They range from synthesis and screening of small peptide libraries to combinatorial approaches utilising phage selection for the *in vitro* evolution of photoswitchable ligands.^32–35^ We selected a more rational design approach, in which the substitution sites were selected based on perceptions of ANP binding to NPR-A from mutational and structural studies.^36–38^ Upon ANP binding, the NPR-A receptor forms a homodimer, where the ligand is buried between the two extracellular domains of the respective monomers. The binding of ANP is asymmetric and one domain interacts mainly with the N-terminal, while the other interacts with the C-terminal part. Important binding interactions involve Phe8, Arg14 and Asn24 of ANP. Phe8 extends to and interacts with a hydrophobic binding pocket, which is critically important for hormonal activity. Arg14 forms hydrogen bonds with both monomers (Asp 62 and Glu119), stabilizing the partially open dimer interface. The C-terminal located Asn24 forms two hydrogen bonds that are important for receptor binding and hormone activity.^38^

Thus, the substitution schemes either involved the direct replacement of the important binding residue, as in the case of N-terminal substitution, or located AMPP close to it, as for the C-terminal and middle substitution schemes. By substituting Phe8 with AMPP, it was envisaged that in these peptides the hydrophobic photoswitch would differentially engage the hydrophobic pocket in its *cis*- or *trans*-form, modifying receptor binding and hormone activity. We dismissed the design of peptides in which AMPP was placed adjacent to Phe8, *i.e.* through substitution of residues Gly9 – Met12, due to the possibility of aromatic stacking. In the peptides with the middle substitution scheme, AMPP was placed adjacent to Arg14, to control the hydrophilic interactions of this residue with the receptor. In the N-terminal substitution scheme, AMPP was placed in the ring alongside Cys23 and the disulfide bridge. In these peptides, isomerisation should not only shift the ring structure, but also alter the binding interactions of Asn24 to domain B of the dimerised NPR-A receptor.

At this point, it is worth noting that initial screening of cGMP accumulation returned a single compound, *i.e.* TOP271, as being the most active and most isomer-dependent peptide. Thus, we focused on characterisation and investigation of this lead compound and further details on the cGMP assays can be found under “optical control of cGMP generation”. Although our design approach was restricted to nine peptides, with many possible patterns being omitted, it led to the isolation of the functional compound TOP271. This not only validates our rational design strategy, but also confirms the targeting of ANP/NPR-A interactions for the control of receptor binding and activation.

### Photophysical properties of AzoANP peptides TOP263-271

**Fig. 2:**
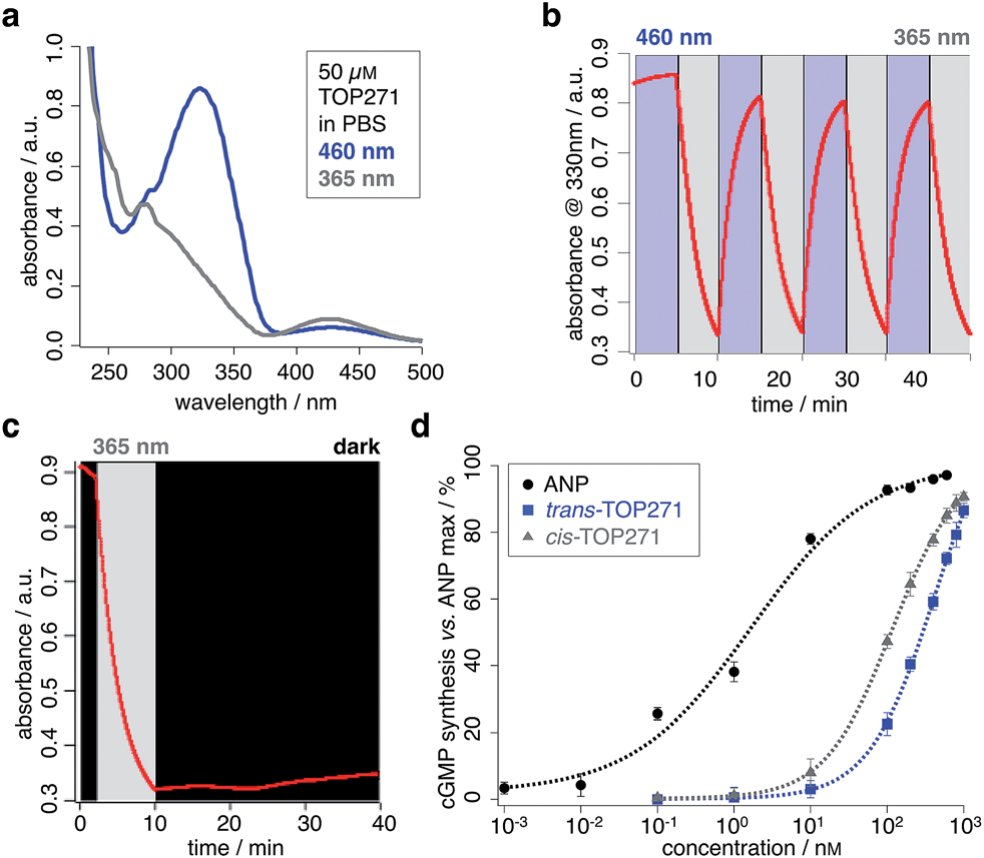
TOP271 photodynamics and cGMP concentration-responses. (a) UV/Vis spectra of *cis*- and *trans*-TOP271. (b) Reversible switching of TOP271 with UV (365 nm) and blue (460 nm) light. (c) Bistability of TOP271 when switched to the *cis*-isomer by means of UV light (365 nm) and subsequently left in the dark. (d) Concentration-response curves of *cis*- and *trans*-TOP271 in NPR-A transfected HEK293T cells assessed by cGMP HTRF, 30 min incubation time. EC_50_ values for ANP = 2.0 ± 0.4 nM, *cis*-TOP271 = 127 ± 11 nM and *trans*-TOP271 = 468 ± 59 nM correspond to two independent assays. Values represent the mean ± SEM.

The photochromic AzoANP peptides including native human ANP (hsANP) were synthesised through solid-phase peptide synthesis (SPPS), characterized by high resolution mass spectrometry, and their purity assessed by reverse-phase HPLC: all were <3.7 ppm of the calculated mass and RP-HPLC revealed high purity (see ESI† for MS and HPLC data). The switching kinetics of all “azologued” peptides were determined by UV/Vis spectroscopy (TOP271 Fig. 2a–c, TOP263-270 ESI Fig. 1–3 and ESI Tables 1 and 2†). Starting with compounds in the dark-adapted state (*vide infra*), a decrease in the π–π* and an increase in the n–π* band was observed in response to UV light (*λ* = 365 nm) (Fig. 2a), with reversion of this switching process in response to blue light (*λ* = 460 nm).

As expected for an electron-poor azobenzene, all peptides were bistable (TOP271 Fig. 2c, TOP263-270 ESI Fig. 3†).¶
¶All peptides are available for academic use from the Hoffmann-Röder lab upon request. This advantageous trait allows samples to be pre-illuminated prior to application rather than needing constant illumination. We determined the thermal *cis* → *trans* relaxation rate *k*_obs_ in PBS buffer. This was performed at room temperature, and because the compounds were bistable, the initial back-relaxation was obtained as a linear function from the first 30 min after ceasing illumination. Peptides TOP263-270 showed *k*_obs_ × 10^–4^ a.u._330 nm_ min^–1^ between 0.28–2.83, with the most potent compound TOP271 being 0.98 a.u._330 nm_ min^–1^ (see ESI Table 2,† compare with *k*_obs_ = 3.58 × 10^–4^ a.u. min^–1^ for unsubstituted azobenzene in benzene at 0 °C).^39^ To examine the structural relations between ANP and both isomers of TOP263-271, CD spectra were recorded in 40% buffered aqueous 2,2,2-trifluoroethanol (TFE) solutions. TFE is needed for the peptides to form stabilised secondary structures instead of random coils,^40^ and the optimal TFE concentration of 40% was determined with ANP in different aqueous buffered mixtures (ESI Fig. 4a†). The spectra of the peptides TOP263-271 showed differences between their *cis*/*trans*-isomers, but remained similar to that of native ANP, with no observable trend (ESI Fig. 4b and c†). TOP271 was subjected to further characterisation for both its *cis*- and *trans*-form by NMR spectroscopy alongside ANP. The spectra were recorded in 35% aqueous TFE-d_3_ solutions to suppress signal broadening and aggregation (see ESI† for NMR data on ANP and *cis*/*trans*-TOP271). The *cis*-TOP271 NMR spectrum showed overlapping signals and thus could not be resolved. Nevertheless, the ^1^H and ^13^C chemical shift values of the *trans*-TOP271 peptide could be unambiguously identified, showing the incorporation of AMPP into the backbone of the peptide and the overall correct structure.

### Optical control of cGMP generation

To assess the most suitable peptide for further analysis, cGMP generation was measured in HEK293T cells transiently transfected with NPR-A.^41^ cGMP is a major effector of cellular metabolism,^42^ with effects on adipose tissue,^43–45^ liver^46,47^ and the brain.^48^ Alongside nitric oxide, the natriuretic peptides are the major potentiators of cGMP generation, with downstream signalling effects on phosphodiesterases (PDEs),^49^ cGMP-dependent proteinkinases (PKGs)^50^ and cyclic nucleotide-gated channels (CNGs).^51^ ANP induces smooth muscle relaxation through increases in intracellular cGMP levels and activation of PKGI, which subsequently leads to a decrease in cytosolic Ca^2+^ levels and reduced Ca^2+^-sensitivity of the contractile system.^12,52,53^ Furthermore, depleting cGMP levels leads to depolarization in rods of the retina, triggering action potentials that transduce signals to perceive light.^54^

To test cGMP synthesis, each peptide was applied as the *trans*- or *cis*-isomer by keeping them either in the dark or pre-illuminating with UV light (*λ* = 365 nm) for 15 minutes, respectively. Using this approach, TOP271, *i.e.* the ANP analogue where AMPP replaces four amino acids at the C-terminal end of the ring, was identified as the most promising candidate due to its highest binding affinity. In addition, a trend in the activity of these azobenzene-containing peptides was revealed: activity towards cGMP synthesis was higher the more amino acids were replaced and the closer their substitution was located to the N-terminus (Fig. 1c). Besides TOP271, only three further peptides from our small AzoANP library, TOP264, –265 and –268, showed NPR-A activation in the μM range (ESI Fig. 5†). The low potency of these compounds and the inactivity of the remaining photochromic ANP peptides (TOP263, –266, –267, –269 and –270) likely stems from the substitution of residues crucial for receptor binding, such as Phe8.^55,56^

With TOP271 as the lead candidate, we attempted to access light-dependent NPR-A activity. The measured cGMP concentration-responses showed that both TOP271 isomers had similar potency to native ANP (EC_50_ = 2.0 ± 0.4 nM) (Fig. 2d), but *cis*-TOP271 (EC_50_ = 127 ± 11 nM) was roughly four times more potent than the *trans*-isomer (EC_50_ = 468 ± 59 nM). Since these EC_50_ values correspond to the maximum incubation time of 30 minutes, we wanted to assess the time-dependency of potency and isomer-biased receptor activation. Therefore, we collected further data with shorter incubation times, which showed fluctuations for the ANP/TOP271 potency difference (ESI Fig. 6, ESI Table 3†). Nevertheless, the difference in potency between *cis*- and *trans*-TOP271 was robust and reproducible.

cGMP competition assays between ANP and *cis*/*trans*-TOP271 showed right-shifted EC_50_ values for ANP, as expected when ligands compete for the same binding site (ESI Fig. 6†). Although cGMP end point assays do not provide direct evidence for ligand-mediated NPR-A over intracellular GC-A activation, this screening shows clear competition. Given the fact that ANP is well characterised to activate NPR-A, we assume that extracellular activation is key to cGMP generation. However, further studies are required to conclusively understand whether TOP271 directly activates GC-A, for instance using FRET-based reporters.

It should also be noted that, although the increase in EC_50_ seems small, signal integration and amplification of cGMP leads to more pronounced responses *in cellulo.* ^57^ With this in mind, we decided to progress TOP271 through to more relevant studies *ex vivo*.

### Optical control of smooth muscle tone and pancreatic beta cell function

**Fig. 3:**
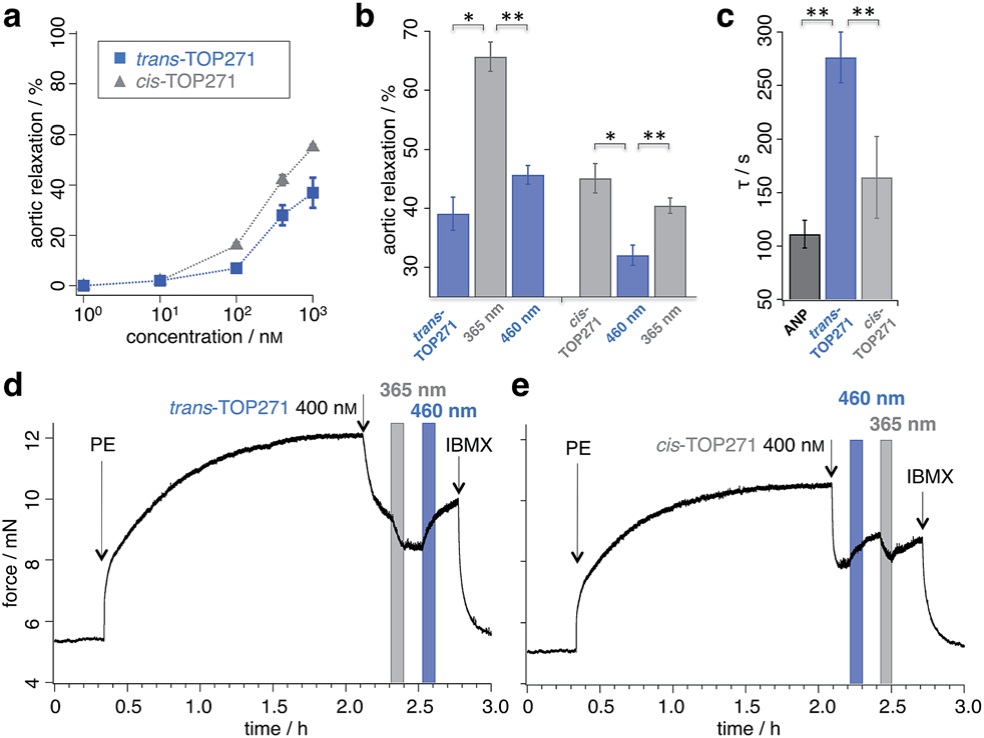
TOP271 allows photocontrol of aortic tissue. (a) Concentration-response curves for *trans*- and *cis*-TOP271. (b) Aortic relaxation for cycles in (d) and (e). (c) *τ* of aortic relaxational responses for 17 nM ANP compared to 400 nM *trans*- and *cis*-TOP271. (d, e) Reversible photocontrol over aortic tension by application of *trans*-TOP271 (d) and *cis*-TOP271 (e) with UV-/blue and blue/UV-light (365 and 460 nm) cycles, respectively. All experiments were conducted in the presence of 200 μM *N*-ω-nitro-l-arginine methyl ester (l-NAME) and 3 μM diclofenac, which were used to block the vascular relaxants nitric oxide and prostacyclin, respectively. PE = phenylephrine was used for initial vasoconstriction, IBMX = 3-iso-butyl-1-methylxanthine was used to induce complete muscular relaxation as an end point control. (*n* = 6 aortic rings from 4 animals) (*P < 0.05, **P < 0.01, repeated measures Student's *t*-test). Values represent the mean ± SEM.

We next sought to address whether TOP271 would allow the optical control of cGMP-dependent processes in a physiologically relevant system, *i.e.* the aortic ring preparation. The treatment of constricted aortic rings with ANP leads to a potent vasodilation, corresponding to the blood pressure reducing effect of ANP.^58^ Accordingly, we predicted that TOP271 would allow reversible, light-controlled vasoactive responses, with *cis*-TOP271 being the stronger effector at specific concentrations. Concentration–response curves were obtained for vasodilation in pre-constricted aortic rings following exposure to pre-illuminated *cis*- (*λ* = 365 nm) and *trans*-TOP271 (*λ* = 460 nm), and showed increased potency for the former isomer in the 100 nM to 1 μM range (Fig. 3a, ESI Fig. 7†). Although this concentration–response indicated a significant difference in receptor activation for 100 nM TOP271, we decided to use 400 nM TOP271 to trigger a stronger isomer-dependent vasodilation. Thus, the application of dark-adapted *trans*-TOP271 led to strong vasodilation, which was enhanced following UV (*λ* = 365 nm) illumination to induce *cis*-isomerisation, and again reversed after blue light (*λ* = 460 nm) exposure to induce *trans*-accumulation (Fig. 3b and d, ESI Fig. 8†).

Conversely, to examine the *cis* → *trans* → *cis* isomerisation cycle, pre-illuminated (*λ* = 365 nm) *cis*-TOP271 was added to the organ bath, leading to a potent vasodilation (Fig. 3b and e). Subsequent *trans*-isomer accumulation by exposure to blue light (*λ* = 460 nm) elicited vasoconstriction, which again could be reversed by UV (*λ* = 365 nm) illumination. Notably, the speed of the initial vasodilation was 1.5× higher for *cis*- compared to *trans*-TOP271, with the former being analogous to ANP (Fig. 3c). Although the experimental setting limited TOP271 switching to three iterations, isomerisation is robust and extended switching cycles are conceivable depending on the application, where degradation and clearance together with internalization and desensitization can play roles in repeated switching efficiency. Still, the multiple switching of TOP271 facilitates the implementation of this compound to induce desired vasoactive effects locally, within a spatially confined area. This clearly sets TOP271 apart from other agonists or inhibitors, which in general only allow for systemic application.

**Fig. 4:**
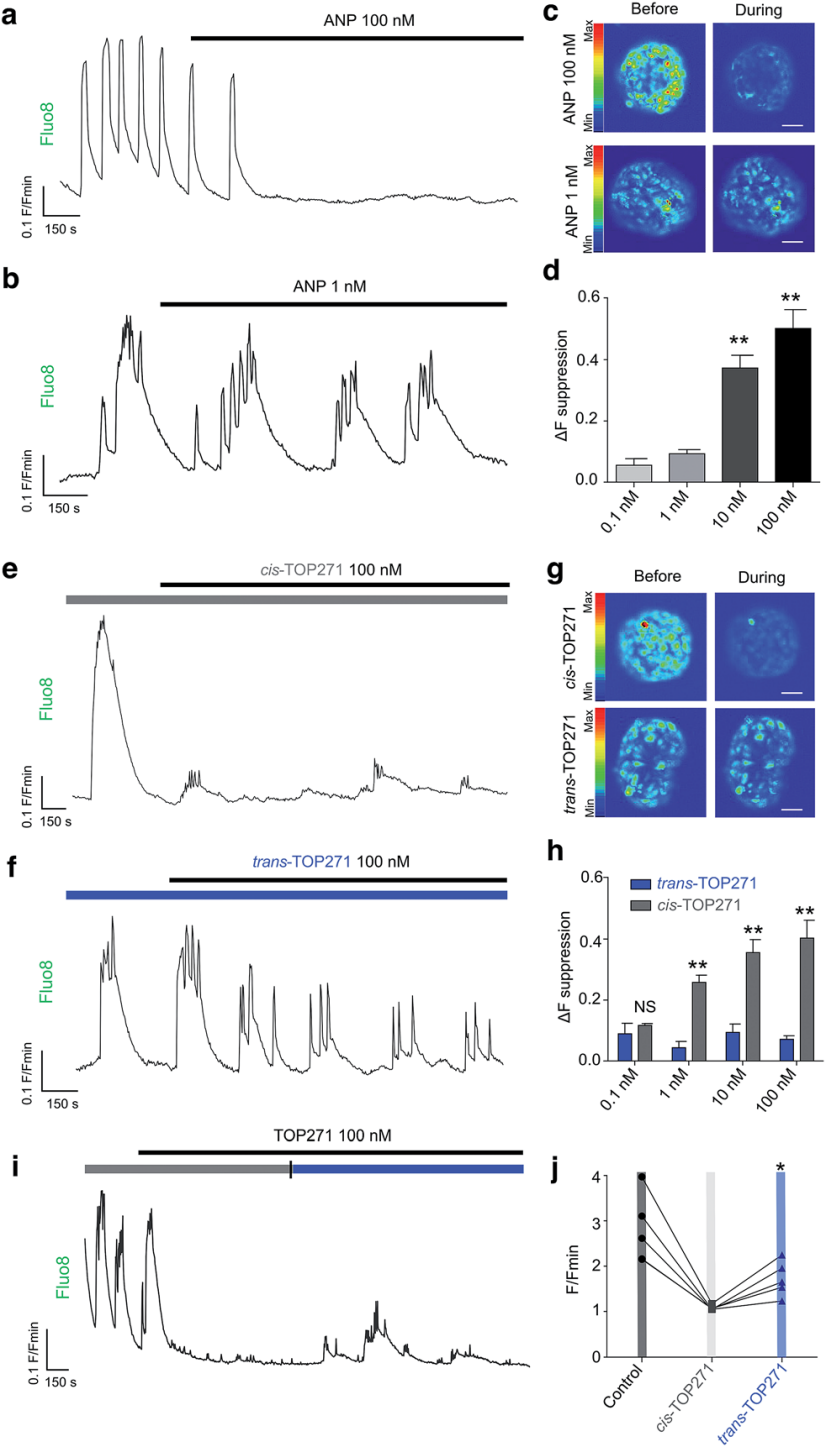
TOP271 allows photocontrol of pancreatic beta cell function. (a–d) ANP concentration-dependently suppresses Ca^2+^ responses in beta cells (representative traces shown) (scale bar 25 μM) (*n* = 4–6 islets for each concentration) (**P < 0.01, one-way ANOVA). (e–h) *cis*-TOP271 concentration-dependently inhibits Ca^2+^ spike amplitude, whereas *trans*-TOP271 is less effective at all concentrations tested (representative traces shown) (scale bar 25 μM) (*n* = 4–6 islets for each concentration) (**P < 0.01, two-way ANOVA). (i and j) TOP271 allows reversible photocontrol of beta cell function, with blue (458–488 nm) light partially restoring Ca^2+^ responses (*n* = 5 islets) (*P < 0.05, repeated measures one-way ANOVA). Values represent the mean ± SEM.

Insulin-secreting pancreatic beta cells express NPR-A, and ANP action is thought to provide a potential explanation for the association between cardiovascular and metabolic dysregulation.^59,60^ We observed that native ANP concentration-dependently (0.1–100 nM) suppressed beta cell function at physiologically-elevated glucose (8 mM) levels, as shown by a reduction in the amplitude of intracellular Ca^2+^ fluxes in intact islets of Langerhans (Fig. 4a–d). These findings could be replicated using UV pre-illuminated (*λ* = 365 nm) *cis*-TOP271 (Fig. 4e–h), which also robustly suppressed Ca^2+^ rises from 1–100 nM. By contrast, dark-adapted *trans*-TOP271 induced only a small decrease in beta cell Ca^2+^ spiking activity (Fig. 4e–h). Reversibility could be achieved by applying *cis*-TOP271 and then illuminating with blue light (*λ* = 458–482 nm) to induce *trans*-isomerisation (Fig. 4i and j). Restoration of beta cell function was only partial (Fig. 4j), however, possibly due to cGMP-mediated sequestration of Ca^2+^ into internal stores such as the endoplasmic reticulum.^61^

Pancreatic beta cells have been shown to express NPR-A, and links exist between ANP and diabetes risk.^62^ Indeed, ANP gene expression is increased in the ventricles of rats with reduced beta cell mass, and ANP levels are elevated during diabetes.^59^ The effects of ANP on beta cell function are complex and controversial. While some studies have shown that ANP decreases Ca^2+^ levels and insulin secretion,^10,24^ others have shown stimulatory effects.^11,63^ This likely reflects differences in the time course of application, preparation under examination (*i.e*. dissociated *vs.* intact islets), stimulation state (*i.e.* low *vs.* high glucose) and concentration. With regards to the latter, we were able to show a bimodal relationship where low doses of ANP preferentially affect Ca^2+^ oscillation frequency without altering amplitude, whereas high doses do the opposite (ESI Fig. 9†). Thus, TOP271 may provide an important tool to allow ANP receptor conformation and signalling to be understood in the context of beta and other cell (dys)function.

### Molecular dynamics simulations of ANP and *cis*/*trans*-TOP271

Atomic-level modelling in explicit solvent was conducted to better understand the structure-activity relationships of isomer-dependent NPR-A activation. We focused on the *in silico* structure of native ANP peptide and *cis*/*trans-*TOP271, both in aqueous solution and bound to the NPR-A receptor. The extra-cellular domains were modelled based on the NPR-A crystal structure (PDB: ; 1t34, in complex with rat ANP (rnANP)),^55^ while the receptor-bound ANP peptide and the *cis*/*trans*-TOP271 isomers where based on the NPR-C crystal structure (PDB: ; 1yk0, in complex with human ANP).^56^ We used the latter for our structural peptide modelling to account for the Met12/Ile12 difference between ANP and rnANP, respectively.

**Fig. 5:**
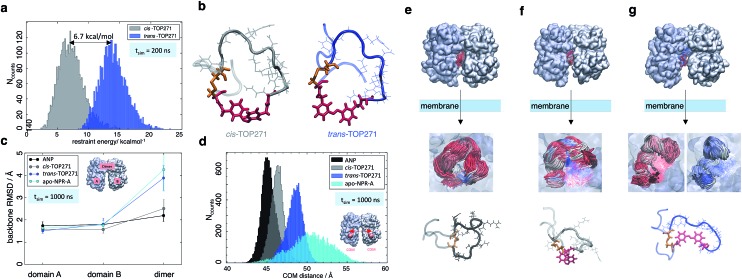
MD simulations of ANP and TOP271. (a) Restraining energy distribution obtained from MD simulations (200 ns) of *cis*- and *trans*-TOP271 in solution, including restraints to keep the sampled conformations close to native ANP (see ESI†). (b) Representative restrained conformations of *cis*- and *trans*-TOP271 illustrating the steric hindrance of *trans*-azobenzene to fit into the native ring structure (red: azobenzene, orange: Cys7-Cys23 disulfide bridge). (c) Mean backbone-Cα RMSD and standard deviation of the NPR-A dimer and the both receptor domains A and B after 1 μs simulation, calculated for bound native ANP (black), bound *cis*/*trans*-TOP271 (gray and blue, respectively) and apo-NPR-A (cyan). The overall receptor RMSD clearly differs between native and *cis*-TOP271 *vs. trans*-TOP271 and apo-NPR-A. (d) Center-of-mass distance (orange balls and arrow) of the membrane-proximal NPR-A domains after 1 μs simulation. For bound native ANP (black) and *cis*-TOP271 (gray) the receptor domains tend to close compared to *trans*-TOP271 and the apo-NPR-A (blue and cyan). (e–g) Representative NPR-A and peptide conformations for bound native ANP (e) and bound *cis*/*trans*-TOP271 (f and g, respectively). Top row: isomer-dependent overall receptor geometry and binding site coverage visualised by time-superposition of the disulfide-connected isomer segment Cys7-Cys23 (red: ≤300 ns, white ≤600 ns, blue ≤1000 ns). Middle row: zoom into the time-superposition illustrates differences in the conformational ensemble of native ANP and *cis*-TOP271 *vs. trans*-TOP271 (azobenzene in line design). Bottom row: representative isomer conformations extracted from the simulated ensemble (red: azobenzene, orange: Cys7-Cys23 disulfide bridge).

In a first step, the *cis*/*trans*-TOP271 isomers were simulated for 200 ns in the absence of the NPR-A receptor. To compare the affinity of both isomers to adopt the bound ANP ring structure, distance restraints with respect to the ANP crystal structure were applied between all Cα-atom pairs within the ring, neglecting the two terminal tails. The resulting restraint energy distributions show a clear difference between the isomers (Fig. 5a), with the energy of *trans*-TOP271 being on average 6.7 kcal mol^–1^ higher. This likely derives from the rigid, extended *trans*-azobenzene structure, which sterically prevents adoption of the native ring structure (Fig. 5b).

To elucidate the effect of the isomeric conformational differences on receptor geometry, unrestrained simulations of 1 μs length were performed for NPR-A bound to *cis*/*trans*-TOP271, ANP and apo-NPR-A. The overall receptor RMSD significantly differs for *cis*- and *trans*-TOP271, with the former matching ANP and the latter being similar to apo-NPR-A (Fig. 5c). These isomer-dependent differences in receptor geometry are related to a change in relative orientation of the two NPR-A dimers: while there are no ligand-dependent orientation changes in the membrane-distal domains (ESI Fig. 10a†), the membrane-proximal domains tend to close around ANP and *cis*-TOP271, but remain more open in the case of *trans*-TOP271 (Fig. 5d–g). This is in agreement with the crystal structures of the different apo- and ligand-bound natriuretic peptide receptors, which show up to 20 Å distance change between the two C-terminal/membrane-proximal receptor domains upon ligand binding.^55,56^ *trans*-TOP271 thereby resembles the apo-form, in which fluctuating membrane-proximal distances shift the receptor towards an open state (Fig. 5c). These changes in receptor geometry can be assigned to isomeric differences in the bound conformation. Whereas the preferred conformations of *cis*-TOP271 are comparable to the crystal ring structure of ANP, the conformational ensemble of *trans*-TOP271 is narrower and more hairpin-like (Fig. 5e–g). The NPR-A-bound crystal structure of ANP also reveals a central pore in the 17 amino acid ring that is essential for ligand binding; only *cis*-TOP271 is able to adapt this donut-like conformation, while the *trans*-isomer forms a closed structure (Fig. 5e–g).

Lastly, we attempted to quantify the twist motion of the NPR-A membrane-proximal domains upon ligand binding, which is thought to initiate intracellular GC activation.^55^ Here, we detected a less prominent isomer dependency for the selected twist angle of the ligand-bound receptor domains compared to apo-NPR-A (see ESI Fig. 10b and ESI Table 3†). While the binding of ANP leads to a focusing of the twist angle distributions in NPR-A, bound *cis*- and *trans*-TOP271 show broader, shifted distributions and the apo-NPR-A inherits large angle fluctuations. This shows on the one hand the higher similarity of angle distributions of ANP and *cis*-TOP271 compared to *trans*-TOP271, but on the other hand also the flexibility of the apo-NPR-A membrane-proximal regions. Notably, crystal structures represent only a structural snapshot, while MD simulations cover a whole ensemble of structures and as a result we conclude that the reduced distance between the membrane-proximal domains in the ligand-bound state, and not the twisting motion of the NPR-A, is the major trigger for receptor activation.

In summary, our MD simulations showed a higher flexibility of the apo-NPR-A and the *trans*-TOP271-NPR-A receptor complex, whereas the membrane-proximal receptor domains tend to close around ANP and *cis*-TOP271. The simulations hence suggest an alternative regulation of guanylyl cyclase activity, in which the binding of ANP and *cis*-TOP271 to NPR-A suppresses dynamic fluctuations of the membrane-proximal domains of both receptor dimers, leading to defined ligand/receptor structures.

## Conclusions

In this study, we present the design, synthesis, evaluation and application of TOP271, a peptidic hormone based on ANP with a photoresponsive azobenzene unit. Acting *via* the NPR-A receptor to generate cGMP, TOP271 allows the reversible photocontrol of contraction/dilation in aortic tissue, as well as Ca^2+^ oscillations in rodent islets of Langerhans. Although photodependent cGMP synthesis was described earlier, these approaches rely on the genetically-encoded photosensitive proteins EROS^64,65^ or BeCyclOps,^66,67^ whereas TOP271 allows unprecedented photocontrolled cGMP synthesis in native tissue. EROS and BeCyclOps are based on bacterial flavin-containing photoreceptors and fungal rhodopsins, respectively, and can be used to induce penile erection in male rats or tactic behavior in *C. elegans* following illumination. A drawback of EROS is the residual cAMP activity, caused by the specific mutation of an adenylyl cyclase to an engineered guanylyl cyclase. BeCyclOps on the other hand utilizes native guanylyl cyclase activity and was shown to specifically engage only cGMP synthesis, but requires genetic introduction. Photochromic ligands like TOP271 avoid these issues and remain exogenously applied, thereby only targeting and activating the protein of interest. It should be noted that the stability of azobenzene compounds introduced *ex vivo* or *in vivo* has to be evaluated on a case-to-case basis as degradation of the diazene unit might occur depending on cell environment or azoreductase expression. While we have shown that this is not the case for polar compounds such as JB253 ^68^ and JB558 ^69^, careful evaluation has to be performed before any clinical applications can be envisioned.

With TOP271, we could selectively and reversibly manipulate the NPR-A/cGMP signalling pathway with high spatio-temporal precision. Interestingly, the 4-fold higher potency of *cis*-TOP271 for cGMP generation detected *in vitro* in transfected HEK293T cells is sufficient to trigger a more pronounced vasodilation *ex vivo* in aortic ring tissue. Although the exact intracellular cGMP concentration is an active source of research efforts, the changes observed are in agreement to prior findings, where small changes in the concentration of this second messenger provoke a significant amplification of downstream signals.^70^ It also showcases the robustness and applicability of TOP271, which we believe will enable precise control of hemodynamic processes, contributing to the dissection of vascular function in health and disease.

Moreover, TOP271 not only demonstrates the successful transformation of ANP into a photoswitchable peptide, but also extends the toolbox of photochromic ligands to all classes of transmembrane receptors. The incorporation of azobenzenes into peptides and proteins has been achieved in a multitude of systems in the last decade, *i.e.* in proteins of *E. coli* ^7,71^ and in short peptides with specific secondary structures such as β-sheet and β-hairpin motifs.^27,29,30,72^ Two major possibilities should be distinguished: (i) having an azobenzene as an amino acid residue and (ii) having an azoswitch in the peptide backbone. While the former has been used to gain optical control over binding affinity of transcription factor and cell adhesion molecules,^71,73^ the latter was successfully applied to the optical control of muscle contraction^31^ and secondary structure formation.^74^ We envision our design herein, together with AzoChig^28^ and LirAzo,^8^ to be highly applicable to all kinds of peptides (*e.g.* neuropeptides, such as oxytocin, vasopressin, kisspeptin), as backbone substitution allows a larger conformational change upon isomerisation and therefore a larger change in affinity and/or efficacy. With recent synthetic efforts in mind,^75^ tetra-*ortho*-chloro-AMPP, exhibiting red-shifted switching wavelengths and high bistability, can be envisioned for the incorporation into target peptides. Such breadth already encompasses hairpin structures, α-helices and now macrocyclic structures, but can potentially be extended to antibodies, immunogens, peptidic hormones and receptors, where fine regulation of protein function by tertiary structure stabilization/destabilization is necessary for function.^76^ Thus, the present findings set the stage for photochromic peptides to become a mainstay for optical control of biological processes using photopharmacology.

## Methods/experimental section

Experimental procedures and chemical characterization can be found in the ESI.† Experimental protocols regarding live animals were approved by the University of Birmingham's Animal Welfare and Ethical Review Body (AWERB) and carried out in accordance with the Animals (Scientific Procedures) Act 1986 of the United Kingdom.
