# Supplementary material for: Optical control of a receptor-linked guanylyl cyclase using a photoswitchable peptidic hormone
Source: Chem Sci. 2017 Apr 19;8(6):4644–53. doi: 10.1039/c6sc05044a (PMC5471452; doi:10.1039/c6sc05044a)
Supplement: Supplementary file 1 [file SC-006-C6SC05044A-s001.pdf]

Electronic Supplementary Material (ESI) for Chemical Science.
This journal is © The Royal Society of Chemistry 2017

# Optical control of a receptor-linked guanylyl cyclase using a photoswitchable peptidic hormone

# Supporting Information

Tom Podewin,[‡,a,b] Johannes Broichhagen,[‡,a,b] Christina Frost,[c] Dieter Groneberg,[d] Julia Ast,[e,f] Helena Meyer-Berg,[a] Nicholas H. F. Fine[e,f], Andreas Friebe,[d] Martin Zacharias,[c] David J. Hodson,[e,f] Dirk Trauner,*[a] and Anja Hoffmann-Röder*[a]

---

a. Department of Chemistry and Center for Integrated Protein Science, LMU Munich, 81377 Munich, Germany.

b. Present Address: Max Planck Institute for medical research (MPImF), Jahnstraße 29, 69120 Heidelberg, Germany.

c. Department of Physics, Technical University of Munich, James-Franck-Str. 1, 85748 Garching, Germany.

d. Julius-Maximilian-University Würzburg, Institute of Physiology, Röntgenring 9, 97070 Würzburg, Germany.

e. Institute of Metabolism and Systems Research (IMSR) and Centre of Membrane Proteins and Receptors (COMPARE), University of Birmingham, Edgbaston, B15 2TT, UK.

f. Centre for Endocrinology, Diabetes and Metabolism, Birmingham Health Partners, Birmingham, B15 2TH, UK.

‡. These authors contributed equally.

*Table of Contents*

**1. Chemistry and Spectroscopy** .......... 3

**1.1. General** .......... 3

**1.2. Peptide synthesis** .......... 4

**1.3. HPLC traces of AzoANP peptides TOP263-271 and ANP** .......... 5

**1.4. Photodynamics of peptides TOP263-271** .......... 6

**1.5. NMR Spectra of ANP and *cis/trans*-TOP271 peptides** .......... 7

1.5.1. ANP .......... 7

1.5.2. *trans*-TOP271 .......... 9

1.5.3. *cis*-TOP271 .......... 9

**1.6. HRMS-ESI of peptides TOP263-TOP271** .......... 10

**2. Material and Methods** .......... 15

**3. Supplementary Figures and Tables** .......... 19

**4. References** .......... 38

# *1. Chemistry and Spectroscopy*

## 1.1. General

**AMPP synthesis**
AMPP was synthesized according to a previously described synthesis[1,2] and spectra were identical to those reported.

**Peptide synthesis**
Peptides were synthesised on a CEM Liberty 1 Peptide Synthesizer with a CEM Discovery Microwave under standard Fmoc-protected solid phase peptide synthesis protocols with standard reagents. More detailed information regarding peptide synthesis, resins, conditions and coupling reagents can be found in chapter 1.2.

**Reversed-phase HPLC**
Analytical RP-HPLC was performed with Jasco devices (PU- 2080 Plus, LG-2080-02-S, DG-2080-53 and MD-2010 Plus) on a Phenomenex Aeris column (C18, 5 µm, 250x4.6 mm). As eluent water/acetonitrile gradients containing 0.1% TFA was used with a 1 mL/min flow rate. Semi-preparative RP-HPLC was performed on Jasco devices (PU-2087 Plus, LG-2080-02-S and UV-2075 Plus) with a Phenomenex Aeris column (C18, 5 µm, 250x20 mm) and the same eluents were used as for RP-HPLC, but with a 20 mL/min flow rate.

**NMR**
The NMR spectra of the peptides were recorded in 35% aqueous 2,2,2-trifluoroethanol-$d_3$ (TFE-$d_3$) solutions with a BRUKER Avance III HD 800 instrument and calibrated to the residual solvent peak of TFE-$d_3$ ($^1H/^{13}C$: 3.88/126.3 in ppm). Although the compounds exhibited bistability during long carbon and 2D experiments, the peptides were first pre-illuminated with UV light (365 nm) to obtain the *cis*-isomer and the NMR tube was then equipped with a fibre optic coupled to a Polychrome V (Till Photonics) monochromator to deliver constant UV irradiation (365 nm) to prevent thermal *cis→trans* relaxation[3]. Thus, spectra could only be obtained without 20 kHz spinning of the probe, accounting for the signal broadening in the spectra of the photochromic *cis*-peptides. Multiplicities are abbreviated as follows: s = singlet, d = doublet, t = triplet, q = quartet, br = broad, m = multiplet. Spectra are reported based on appearance, not on theoretical multiplicities derived from structural information.

**HRMS**
High-resolution electrospray ionization (ESI) mass spectra were recorded on a Varian MAT 711 MS instrument operating in either positive or negative ionization modes.

**UV/Vis**
UV/Vis spectra were obtained on a Varian Cary 50 Bio UV-Visible Spectrophotometer. The probes were measured in Helma SUPRASIL precision cuvettes (10 mm light path) and irradiated with a Polychrome V (Till Photonics) monochromator.

**CD**

CD measurements were obtained on a Jasco 810 instrument connected to a Jasco CDF-4265 Peltier-Element. The used solvents were either phosphate buffer (20 mM, pH = 5.5) or different dilutions of phosphate buffer (20 mM, pH = 5.5) with TFE. Temperatures were kept at 5 °C unless otherwise stated. Hellma® 1 mm cuvettes where used to measure the samples. As baseline correction a pure buffer spectrum was subtracted from the sample spectrum. Sample concentrations were calculated with the specific absorption at 323 nm, with $\varepsilon_{azobenzene} = 25{,}000\ L\ mol^{-1}cm^{-1}$. The recorded spectra were processed with WaveMetrics Igor 6.37 and were smoothed with a 21-point Savitzky-Golay filter.

## 1.2. Peptide synthesis

As solid-phase a pre-loaded Fmoc-Tyrosine-Wang LL resin (Novabiochem®) with a loading 0.32 mmol/g amino acid was used. The scale of the peptide synthesis was 0.1 mmol using the coupling reagents HBTU/HOBt·$H_2O$ 0.5 M in DMF and DIPEA 2 M in NMP. Fmoc-protected amino acids (Orpegen Pharma and Sigma) with standard residual protecting groups were coupled with a fivefold excess (2 M solutions). Fmoc deprotection was achieved by treatment with 20% piperidine in DMF. The AMPP building block was coupled with only 1.5-fold excess and under usage of the more reactive coupling reagents HATU/HOAt (1.5 eq.) 0.5 M in DMF and NMM (5 eq.) in DMF (2 steps: 1. 300 s, 0 W, r.t.; 2. 1800 s, 23 W, 75 °C). Specified coupling conditions are listed in Supplementary Table 1. After completion of all coupling steps, the resin-bound peptide was transferred into a Merrifield reactor with subsequent global deprotection using a TFA/phenol//$H_2O$/tri*iso*propylsilane (88:5:2:5) cleavage cocktail within 2 h under argon atmosphere. The solution was filtered and the filtrate was precipitated in 180 mL chilled diethyl ether and stored overnight at -38 °C to complete precipitation. The precipitated peptide was centrifuged and after removal of the supernatant the residue was dried, stirred in 25% aqueous DMSO solution at room temperature overnight to ensure disulfide bond formation and purified by RP-HPLC to afford the desired peptide.

Peptide sequences:

| | |
|---|---|
| ANP: | SLRRS SCFGG RMDRI GAQSG LGCNS FRY |
| TOP263: | SLRRS SC **AMPP** G RMDRI GAQSG LGCNS FRY |
| TOP264: | SLRRS SCFGG RMDRI **AMPP** QSG LGCNS FRY |
| TOP265: | SLRRS SCFGG RMDRI GAQSG **AMPP** CNS FRY |
| TOP266: | SLRRS SC **AMPP** RMDRI GAQSG LGCNS FRY |
| TOP267: | SLRRS SCFGG RMDR **AMPP** QSG LGCNS FRY |
| TOP268: | SLRRS SCFGG RMDRI GAQS **AMPP** CNS FRY |
| TOP269: | SLRRS SC **AMPP** MDRI GAQSG LGCNS FRY |
| TOP270: | SLRRS SCFGG RMD **AMPP** QSG LGCNS FRY |
| TOP271: | SLRRS SCFGG RMDRI GAQ **AMPP** CNS FRY |

### 1.3. HPLC traces of AzoANP peptides TOP263-271 and ANP (ANP)

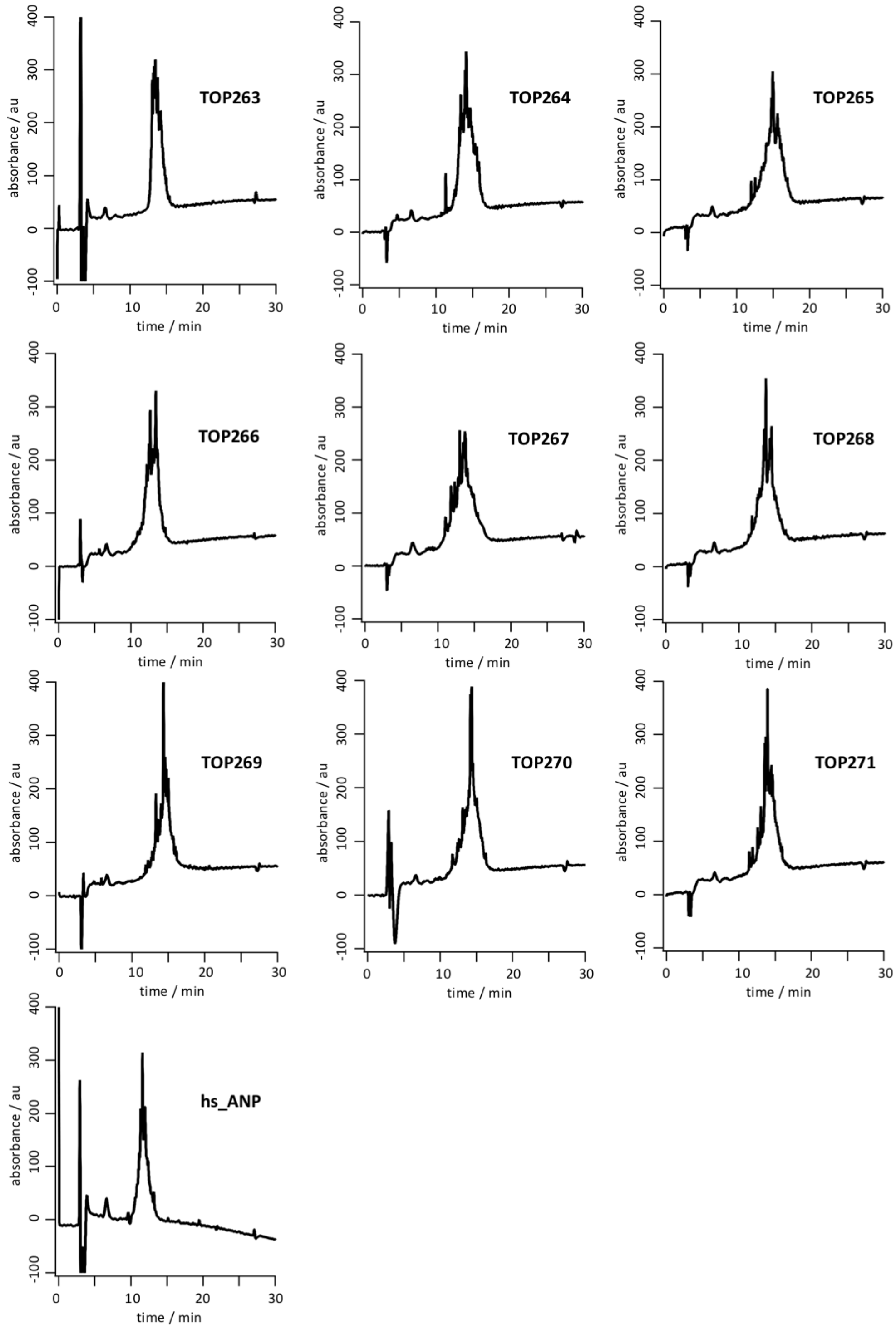


Above are analytical HPLC traces of TOP263-271 and ANP using a gradient MeCN/$H_2O$ = 5/95 → 80/20 over 40 min.

## 1.4. Photodynamics of peptides TOP263-271

*cis*/*trans*-isomerization was characterized by UV/Vis spectroscopy by detection of the π-π*-band (330 nm) and double-exponential fitting of the slopes (WaveMetrics Igor v6.37). τ-Values of *cis/trans*-isomerization and the rate $k_{obs}$ of the thermal back-relaxation are given in Supplementary Tables 1 and 2 and represented in Supplementary Figures 2 and 3.

## 1.5. NMR Spectra of ANP and *cis/trans*-TOP271 peptides

### 1.5.1. ANP

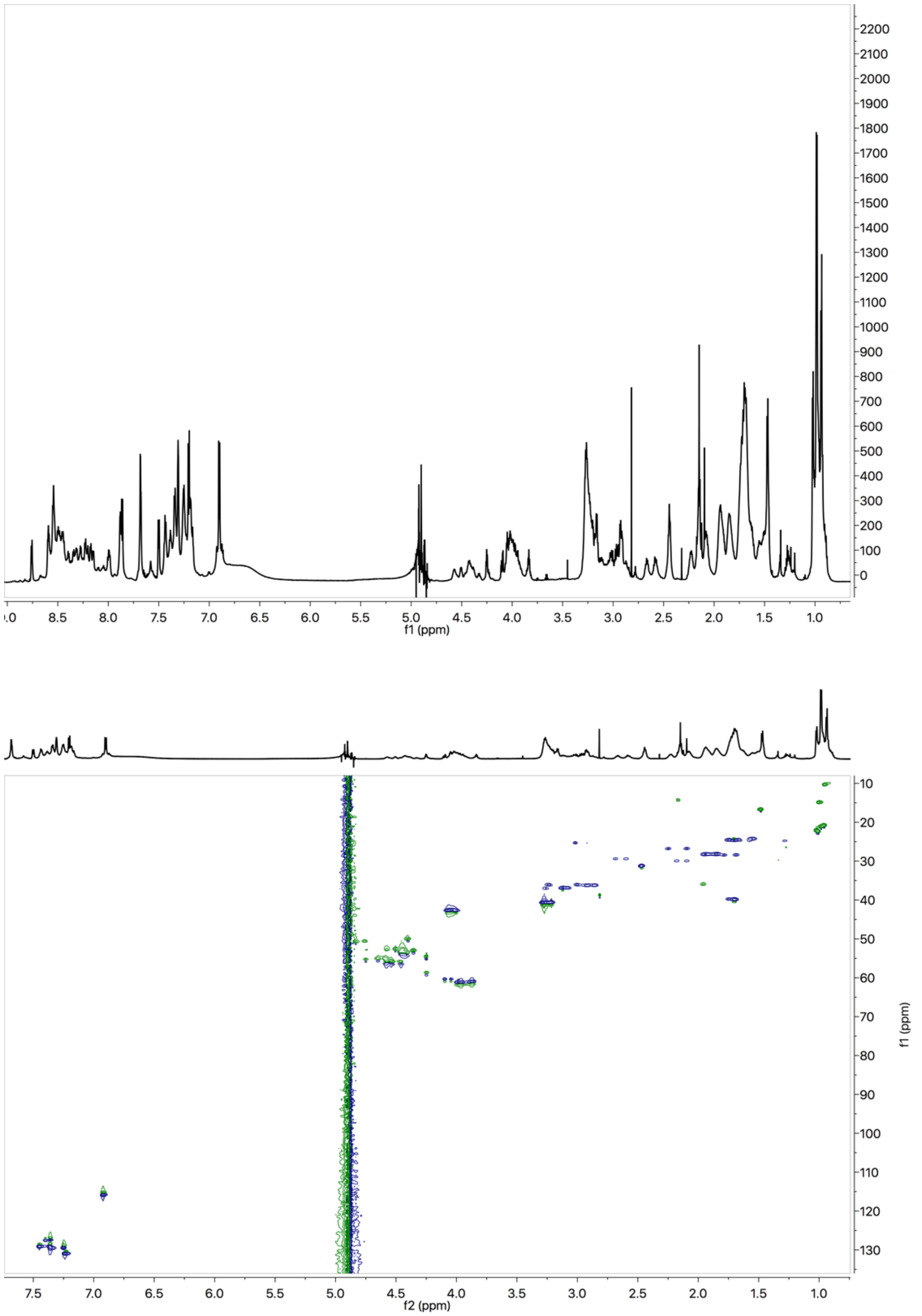


**$^1$H**- (top) and **HSQC**- (bottom) NMR spectra of ANP peptide.

### 1.5.2. *trans*-TOP271

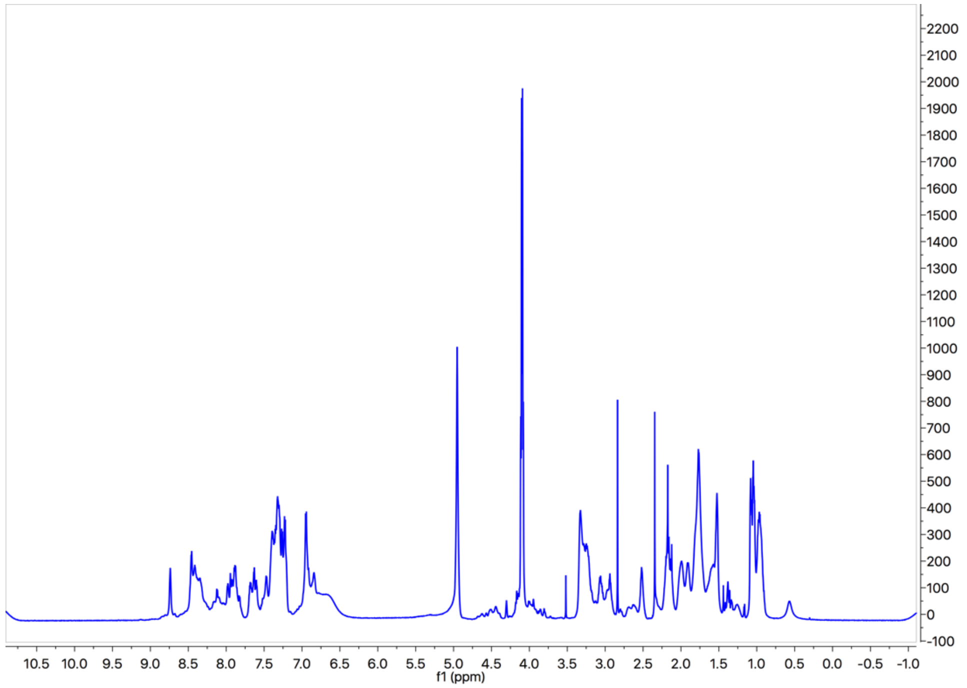


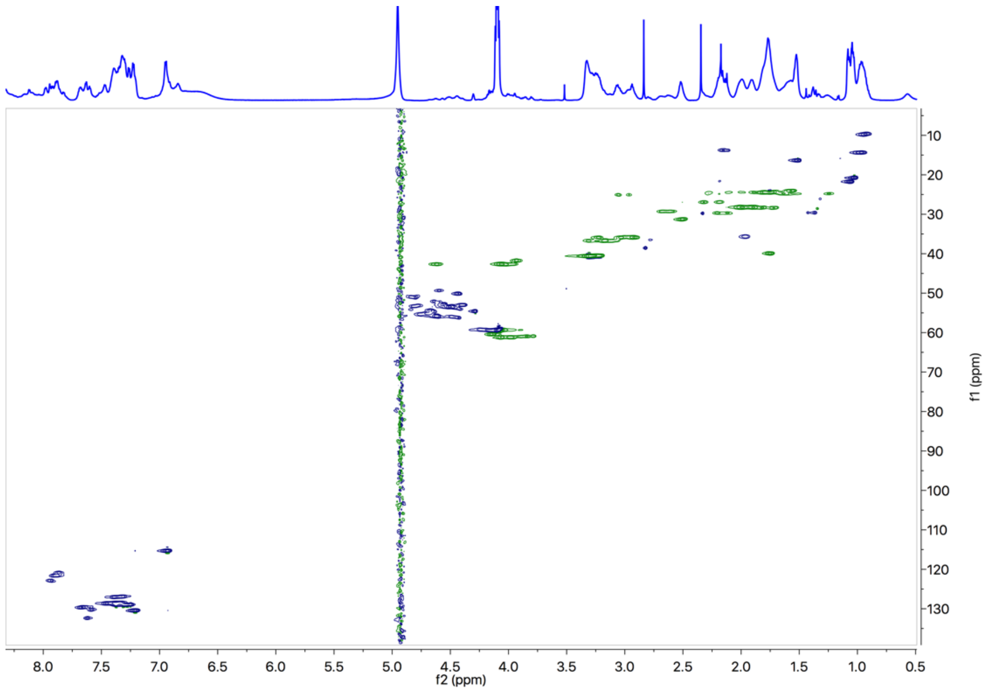


[1]**H**- (top) and **HSQC**- (bottom) NMR spectra of *trans*-TOP271 peptide.

### 1.5.3. *cis*-TOP271

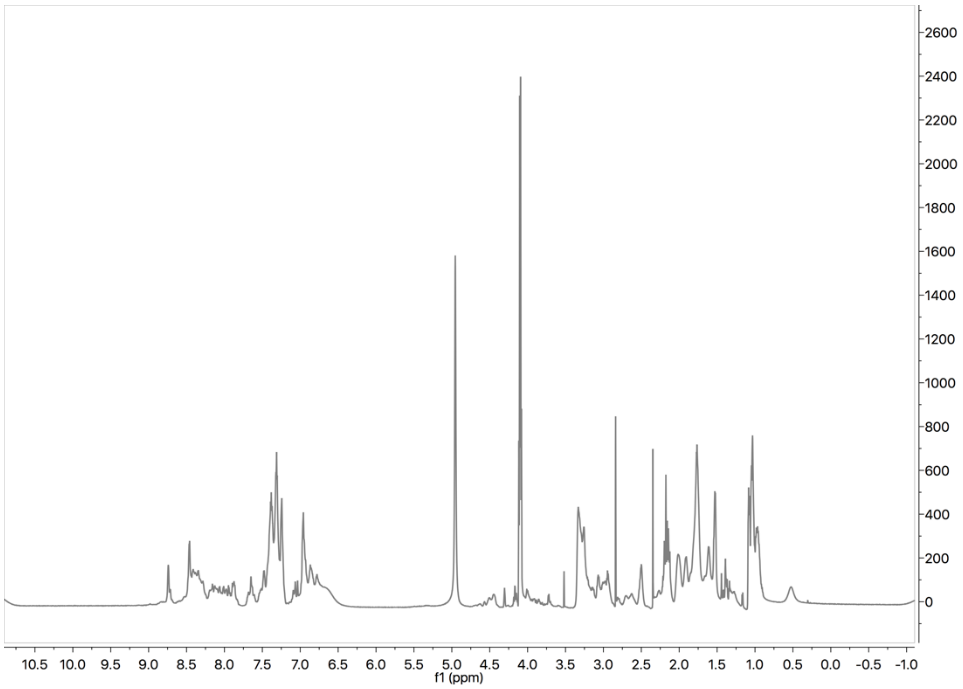


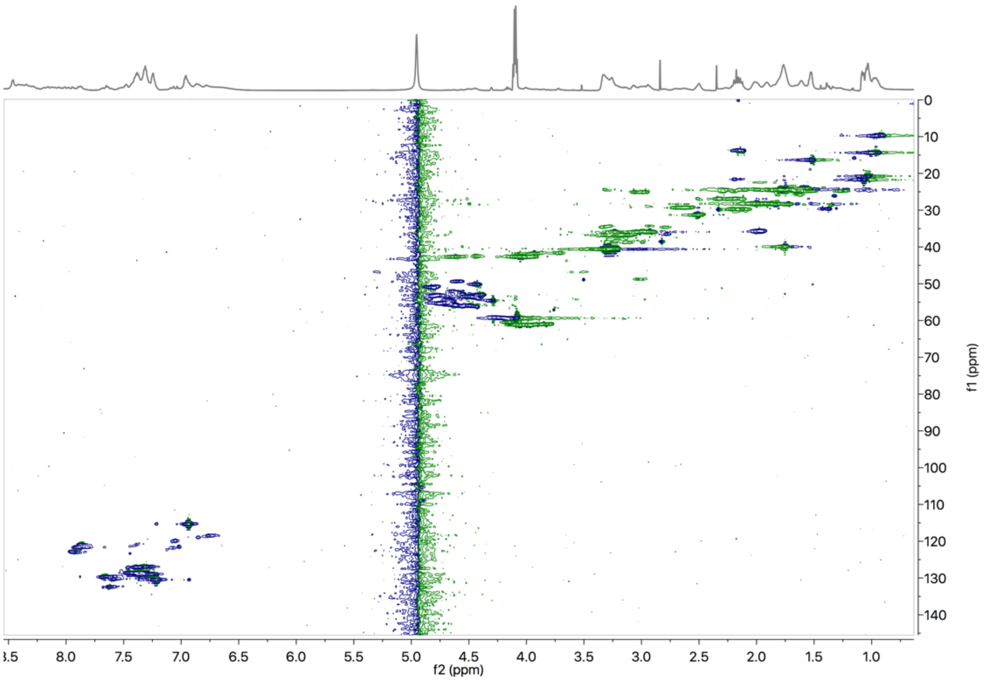


$^{1}$**H**- (top) and **HSQC**- (bottom) NMR spectra of *cis*-TOP271 peptide.

## 1.6. HRMS-ESI of peptides TOP263-TOP271

| Peptid | calc. mono isotopic mass | found mono isotopic mass | Δm/m (ppm) |
|---|---|---|---|
| **ANP** | $[M+4H]^{4+}$ = 3084.4804 | 3084.4858 | -1.75 |
| **TOP263** | $[M+4H+H_2O+3MeCN]^{4+}$ = 3273.5980 | 3273.5979 | 0.02 |
| **TOP264** | $[M+5H-CH_4N_3]^{5+}$ = 3148.4786 | 3148.4780 | 1.87 |
| **TOP265** | $[M+4H+Na]^{5+}$ = 3190.5397 | 3190.5281 | 3.68 |
| **TOP266** | $[M+2H+2NH_4+Na]^{5+}$ = 3148.5192 | 3148.5225 | -1.08 |
| **TOP267** | $[M+5H-NH]^{5+}$ = 3079.4411 | 3079.4391 | 0.70 |
| **TOP268** | $[M+5H-NH]^{5+}$ = 3093.4447 | 3093.4495 | 0.97 |
| **TOP269** | $[M+4H]^{4+}$ = 2918.3840 | 2918.3774 | -2.29 |
| **TOP270** | $[M+5H-H_2O]^{5+}$ = 2948.3868 | 2948.3840 | 2.13 |
| **TOP271** | $[M+5H-H_2O]^{5+}$ = 3003.4300 | 3003.4323 | 2.25 |

## 2. *Materials and Methods*

### cGMP assay in transiently transfected HEK293t cells

HEK293t cells (obtained from the Leibniz-Institute DSMZ, Braunschweig, Germany, #305, regularly mycoplasma tested) were transfected with pCMV5_GC-A [4] and YFP using Lipofectamine 2000® (Life Science, Indianapolis, IN, USA) at >80% confluency in a T25 flask according to the manufacturer's instructions. After 24 h, cells were checked by means of YFP fluorescence for successful transfection (>70% efficiency), washed once and harvested with 1 mL Hepes-Krebs-Ringer (HKR) buffer (containing in mM: 135 NaCl, 5 KCl, 1 $MgSO_4$, 0.4 $K_2HPO_4$, 5.5 D-glucose, 20 HEPES, 1 $CaCl_2$, pH = 7.4) supplemented with PDE inhibitors IBMX (500 µM, Tocris, Bristol, UK, #2840) and Ro 20-1724 (100 µM, Tocris #0415) from a 50 and 10 mM stock in DMSO, respectively. 7.5 µL of cell solution was pipetted into each well of a 384-well plate (white, flat bottom, Greiner, Frickenhausen, Germany). Lyophilized peptides were dissolved in $dH_2O$ at a concentration of 0.5 mM and diluted in HKR to a dilution series with appropriate concentrations. Stocks were allowed to equilibrate for 1 h. *Trans*-peptides were kept in the dark while *cis*-peptides were isomerized by irradiation with a Polychrome V (Till Photonics, Gräfelfing, Germany) monochromator at 350 nm for 15 min. 2.5 µL of the peptidic solutions were added to the cells and *trans*-peptides were kept in the dark while *cis*-peptides were further illuminated with a handheld UV lamp ($\lambda$ = 366 nm) (Camag, Muttenz, Switzerland). The resulting assays were incubated for 30 min before they were lysed and treated with a cGMP HTRF kit (Cisbio, Codolet, France) according to the manufacturer's instructions. cGMP levels were read with a Omega platereader (BMG Labtech, Offenburg, Germany) after 1 h of incubation time and normalized according to the cGMP standards. All experiments were performed in two independent trials in triplicates. The maximum level of activation by 600 nM ANP served as positive control for maximal cGMP generation, cGMP served as positive control for assay functionality, while non-treated cells served for normalization and mock cells as negative control.

### Simulation models

The ligand-binding NPR-A domain was modelled based on the crystal structure of Ogawa *et al.*, neglecting the associated *N*-acetyl-D-glucosamine groups (pdb: 1t34, resolved residues: 1-426 for monomer A and 1-425, except 253-256, for monomer B)[5]. The missing residues 253-256 in monomer B were rebuilt and the two monovalent chloride ions within the membrane-distal domains identified in the crystal structure were included into the simulations. The crystal structure of the receptor-bound ANP ring (pdb: 1yk0) with the disulfide bridge between Cys7-Cys23 was fitted into the binding site of NPR-A (pdb: 1t34). Then, the partially unresolved, disordered peptide tails (residues 1-6 and 24-28) were rebuilt for the simulation in free solution based on a Cα-atom-resolved NMR solution structure (pdb: 1anp, with similar tail sequence as in ANP except for exchanged Asp3 *vs.* Arg3) and for simulation in the NPR-A binding site by linearly adding the missing residues. The *cis/trans*-TOP271 isomers were generated by replacing amino acids Ser19-Gly20-Leu21-Gly22 by azobenzene in either *cis*- or *trans*-conformation. The azobenzene parametrization was calculated using the Antechamber and GAFF Toolkit, version 1.27[6,7].

**Simulation protocol**

All simulations were performed in explicit solvent with 123 mM NaCl at 300 K, in order to match experimental conditions and were performed using the Amber14 simulation package in combination with the all-atom ff14SB force field[8]. The model structures were solvated in an octahedral simulation box filled with explicit TIP3P water molecules[9]. The minimum distance between the solute and the box boundaries was chosen to be 13 Å in case of the flexible TOP271 peptide in solution and 10 Å in case of the bound complex. This results in system sizes of ~11,000 atoms for TOP271 in solution and ~80,000 atoms for the bound complex. In agreement with the experiment, 123 mM of NaCl ions were added to the solvent, including neutralizing counterions. Standard ionization states at pH = 7 were used for ionizable sidechains and periodic boundary conditions were applied and along with long-range Coulomb interactions were treated using the particle-mesh Ewald method using a real-space cutoff of 9.0 Å[10].

Before the production simulation, each system was energy-minimized for 10,000 steps using the steepest-descent algorithm. The system was then gradually heated up to the target temperature of 300 K in steps of 50 K and 40 ps each. A subsequent two-step equilibration was performed using the Berendsen weak-coupling scheme[11], consisting of 2 ns in the NVT ensemble at 300 K with a coupling time constant of $\tau_T = 0.1$ ps and further 2 ns in the NPT ensemble at 300 K and 1 bar with a coupling constant of $\tau_p = 1.0$ ps. During all equilibration steps, harmonic position restraints were applied to backbone Cα-atoms with a force constant of 0.5 kcal $mol^{-1}Å^{-2}$). The production simulations were performed in the NVT ensemble at 300 K and the equations of motions were integrated with a standard leapfrog algorithm. The SETTLE algorithm for bond constraining in combination with hydrogen mass repartitioning allowed for the use of an integration time step of 4 fs[12,13]. In one set of simulations, conformational differences between the isomers *cis*- and *trans*-TOP271 were quantified by applying distance restraints between Cα-atom pairs with reference distances derived from the energy-minimized ANP structure. These simulations were performed in aqueous solution in the absence of the receptor. The harmonic restraints included all Cα-atom pairs within the disulfide-connected segment Cys7-Cys23 except for directly neighboring Cα atoms, resulting in a total of 66 restraints. The restraint force constant was set to 2.0 kcal $mol^{-1}$ $Å^{-2}$.

**Simulation analysis**

The simulated trajectories were analyzed using *Cpptraj*, version 14.05[14]. For the restrained *cis/trans*-TOP271 peptide in solution, the first 10 ns were discarded for equilibration, while a longer equilibration time of 50 ns was issued for receptor-isomer complexes. The overall RMSD of the receptor dimer (residue 1-851) and the single domains (residue 1-426 and 427-851) was calculated for all backbone Cα atoms after fitting each trajectory frame onto the crystal reference structure 1t34[5].

To compare ligand-dependent changes in the membrane-proximal and membrane-distal receptor geometry, each domain was divided into two subdomains (membrane-distal: residue 1-129 plus 285-374, membrane-proximal: residue 130-284 plus 375-426). The opening or closing of the membrane-distal and membrane-proximal receptor geometry was calculated based on the center-of-mass distance of the respective subdomains (see Fig. 3d and SI Fig. 9). The membrane-proximal twist angle was calculated by defining a

center-of-mass vector between the two membrane-proximal subdomains (see SI Fig. 10). The twist was then defined as the angle between the time-dependent vector fluctuations and the corresponding vector in the crystal reference structure 1t34[5]. Simulated conformations were visualized using VMD, version 1.9.2[15]. For each simulation, representative conformations were selected based on the histogram maximum of the respective order parameter of interest (*e.g.* restraint energy, subdomain distance).

**Aortic tensometry**

Murine thoracic aortic rings were mounted in a myograph as described recently[16]. In detail, thoracic aortas from C57BL/6 mice were explanted and cut into rings and mounted on fixed segment support pins in two four-chamber Myograph 610 (Danish Myo Technology, Aarhus, Denmark) containing 5 ml of Krebs–Henseleit solution (containing in mM: 118 NaCl, 4.7 KCl, 2.5 $CaCl_2$, 1.2 $KH_2PO_4$, 1.2 $MgSO_4$, 25 $NaHCO_3$, 7.5 D-glucose, pH 7.4), bubbled with 95% $O_2$ and 5% $CO_2$. Resting tension was set to 5 mN in the presence of Diclofenac (3 μM) and *N*-nitro-L-arginine methyl ester (L-NAME) (200 μM). After equilibration (45 minutes at 37 °C), rings were pre-contracted with 1 μM phenylephrine (PE). Since aortic ring myography is sensitive to temperature fluctuations, effects of direct and indirect illumination on vasoactivity were determined. While direct exposure of the tissue to light provoked a strong, reversible dilatational effect (~70-80%) for both UV (λ = 365 nm) and blue (λ = 460 nm) light, indirect illumination of the surrounding organ bath solution evoked only a minor, reversible tissue dilation (~8-14%) (see SI Fig. 12). The vasorelaxing effects of pre-illuminated *cis*- and dark *trans*-TOP271 were assessed once a steady plateau was reached. Stock solutions of ANP and TOP271 were prepared (10 mM in water), of which an aliquot of TOP271 was either pre-illuminated at λ = 365 nm using a UVP 3UV benchtop UV lamp to obtain the *cis*-isomer, or kept in the dark to maintain the *trans*-isomer. For reversible vasoconstriction/-dilation an optical fibre powered by a UHP-LED 460 nm and a MIC-LED 365 nm (Prizmatix, Givat-Shmuel, Israel) was used to induce *cis*/*trans*-isomerisation of TOP271. Read-out was conducted in between illumination cycles as soon as a steady plateau was reached to exclude any light dilational effects. IBMX (100 μM) was applied to induce maximal relaxation at the end of each experiment. Each experiment was performed in parallel with 2 to 4 aortic rings from 4 animals.

**Islet isolation**

CD1 mice were maintained in a specific pathogen-free facility under a 12 h light–dark cycle with *ad libitum* access to water and food. Animals were euthanized by cervical dislocation and islets isolated using collagenase digestion. Islets were cultured overnight in Roswell Park Memorial Institute (RPMI) medium supplemented with 10% foetal calf serum and 100 U/mL penicillin and 100 μg/mL streptomycin. No randomization was used for animal experimentation, since mice were only used as tissue donors. Experimental protocols were approved by the University of Birmingham's Animal Welfare and Ethical Review Body (AWERB) and carried out in accordance with the Animals (Scientific Procedures) Act 1986 of the United Kingdom.

**Calcium imaging**

Islets were loaded for 40 min with Fluo8 (AAT Bioquest, Sunnyvale, CA, USA), before imaging using a Crest X-Light spinning disk head coupled to a Nikon Ti-E automated base and 10 x/0.4 NA objective. Pulsed excitation was delivered at $\lambda$ = 458–482 nm (400 ms exposure; 0.33 Hz) via a Lumencor Spectra X Light Engine, and emitted signals detected at $\lambda$ = 500–550 nm using a Photometrics Evolve Delta 512 EMCCD. Illumination to induce photoswitching was performed using either continuous exposure to $\lambda$ = 458–482 nm or $\lambda$ = 382–407 nm. In all cases, HEPES-bicarbonate buffer was used, containing in mM: 120 NaCl, 4.8 KCl, 24 $NaHCO_3$, 0.5 $Na_2HPO_4$, 5 HEPES, 2.5 $CaCl_2$, 1.2 $MgCl_2$, 8 D-glucose. $Ca^{2+}$ traces were normalized as $F/F_{min}$ (F = fluorescence at any given timepoint; $F_{min}$ = minimum fluorescence) and relative change in this value over time denoted by a scale bar.

**Statistics**

Non-multifactorial comparisons were made using Student's t-test. Multifactorial comparisons were prepared using one-way ANOVA. Log-transformed concentration-response curves were fitted using non-linear regression and the Hill equation. All analyses were conducted using Graphpad Prism (Graphpad software) and IgorPro (WaveMetrics software), and results deemed significant * at $P<0.05$ or ** at $P<0.01$.

## 3. Supplementary Figures and Tables

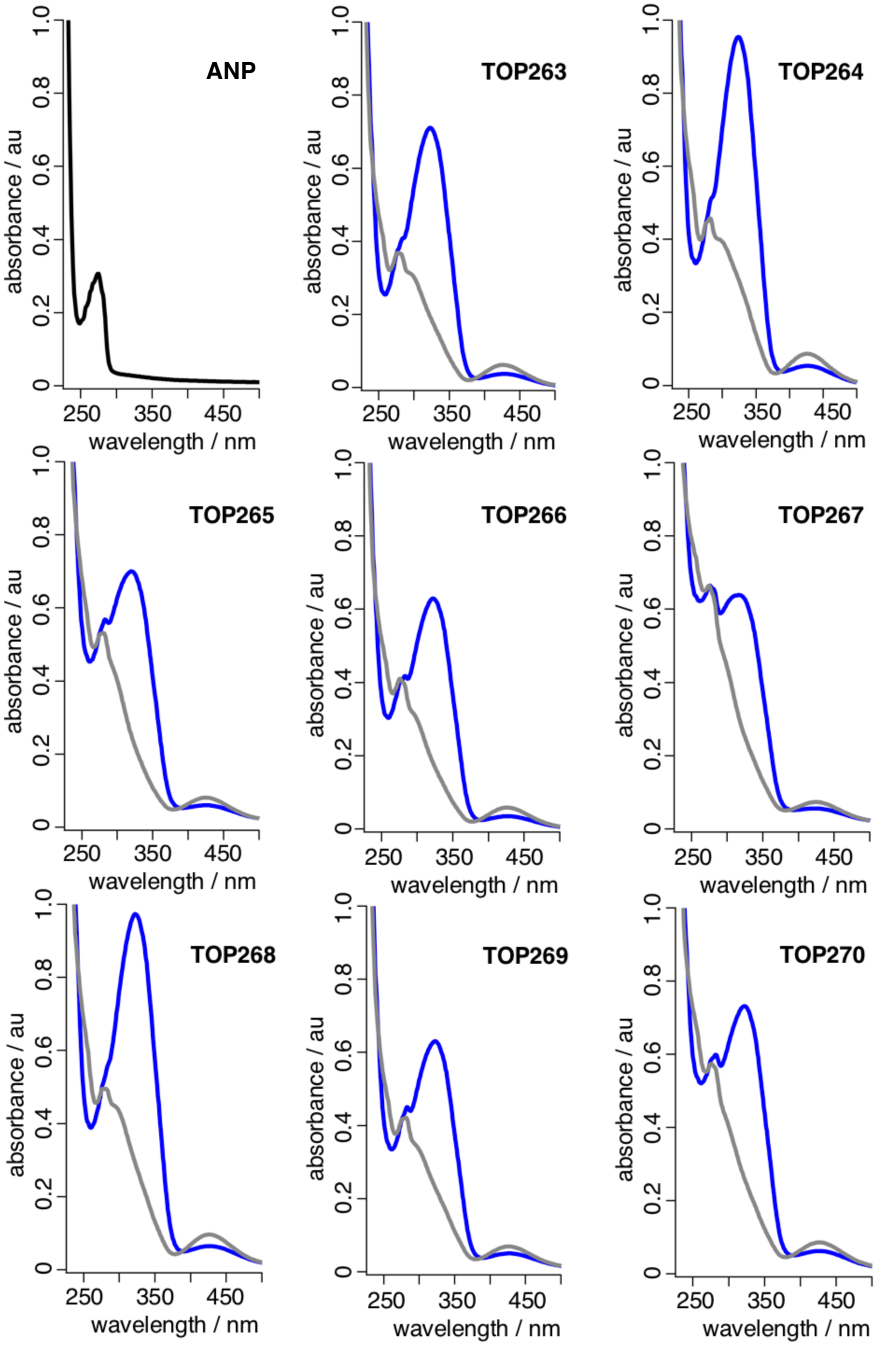


**Supplementary Figure 1:** UV/Vis spectra of ANP (black) and the *cis*- (gray) and *trans*-isomers (blue) of peptides TOP263-TOP270 in PBS buffer at approx. 50 $\mu$M conc.; 25 °C.

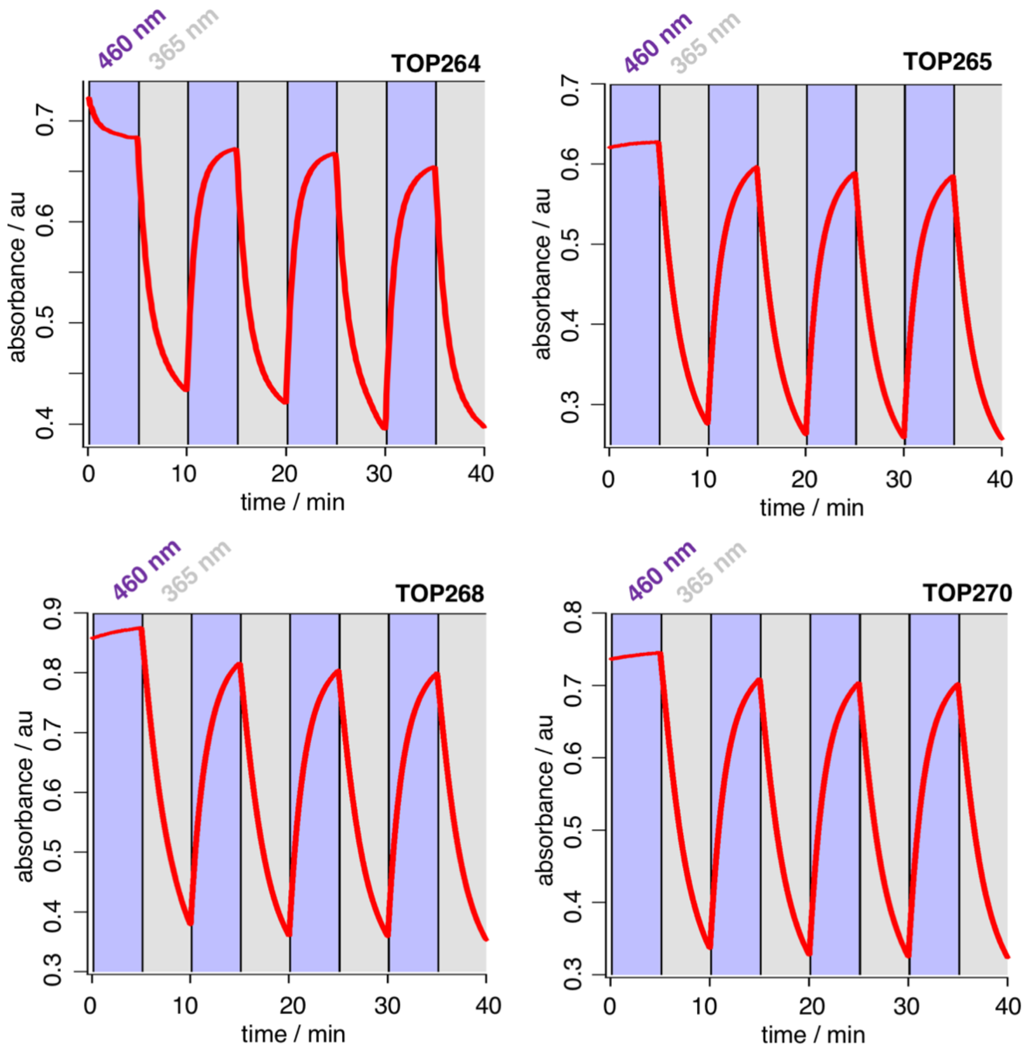


**Supplementary Figure 2:** Representative UV/Vis switching kinetics of peptides TOP263, -264, -268 and -270 measured at 330 nm; PBS buffer, 50 $\mu$M conc.; 25 °C. Reversible *cis*↔*trans*-isomerization was recorded by 365 (*trans*→*cis*) and 460 nm (*cis*→*trans*) illumination cycles (5 min). $\tau$-Values of the *cis/trans*-isomerization of peptides TOP263-TOP271 can be found in Supplementary Table 1.

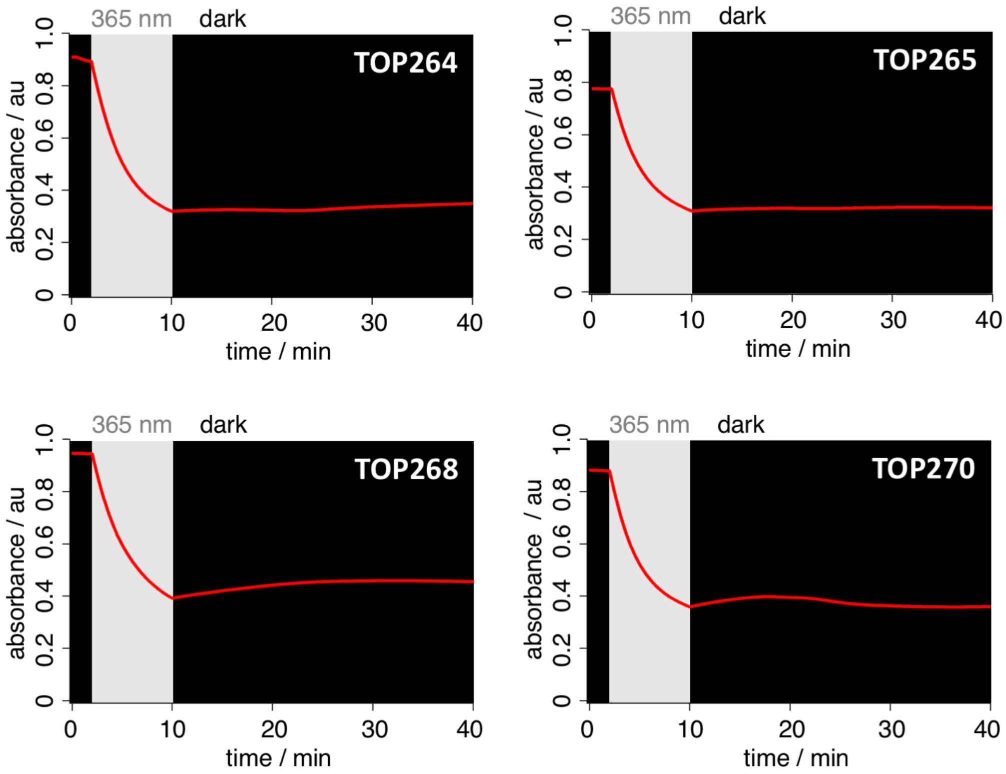


**Supplementary Figure 3:** Representative UV/Vis measurements of the thermal back-relaxation of the *cis*-isomers of peptides TOP263, -264, -268 and -270 at 330 nm in PBS buffer; 25 °C. The rate $k_{obs}$ of the thermal back-relaxation of peptides TOP263-TOP271 can be found in Supplementary Table 2.

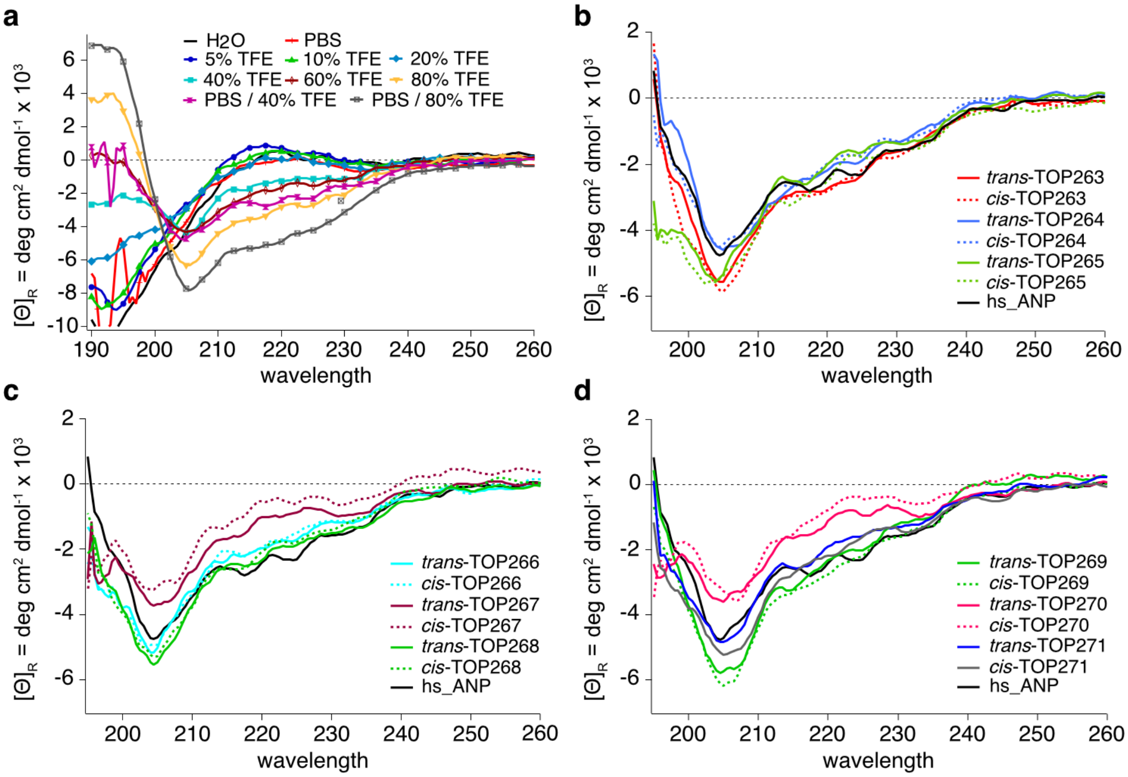


**Supplementary Figure 4:** (a) CD spectra of ANP peptide in $H_2O$, 20 phosphate buffer pH 5.5 and aqueous and buffered TFE (5-80%) solutions. Raising rates of TFE lead to stabilization of secondary structure domains and increase the peptide helical content, with a maximum at 80% aqueous/buffered TFE solutions. (b) CD spectra of the *cis/trans*-isomers of peptides TOP263, TOP264 and TOP265 with two amino acids substituted by AMPP. (c) CD spectra of the *cis/trans*-isomers of peptides TOP266, TOP267 and TOP268 with three amino acids substituted by AMPP. (d) CD spectra of the *cis/trans*-isomers of peptides TOP269, TOP270 and TOP271 with two amino acids substituted by AMPP.

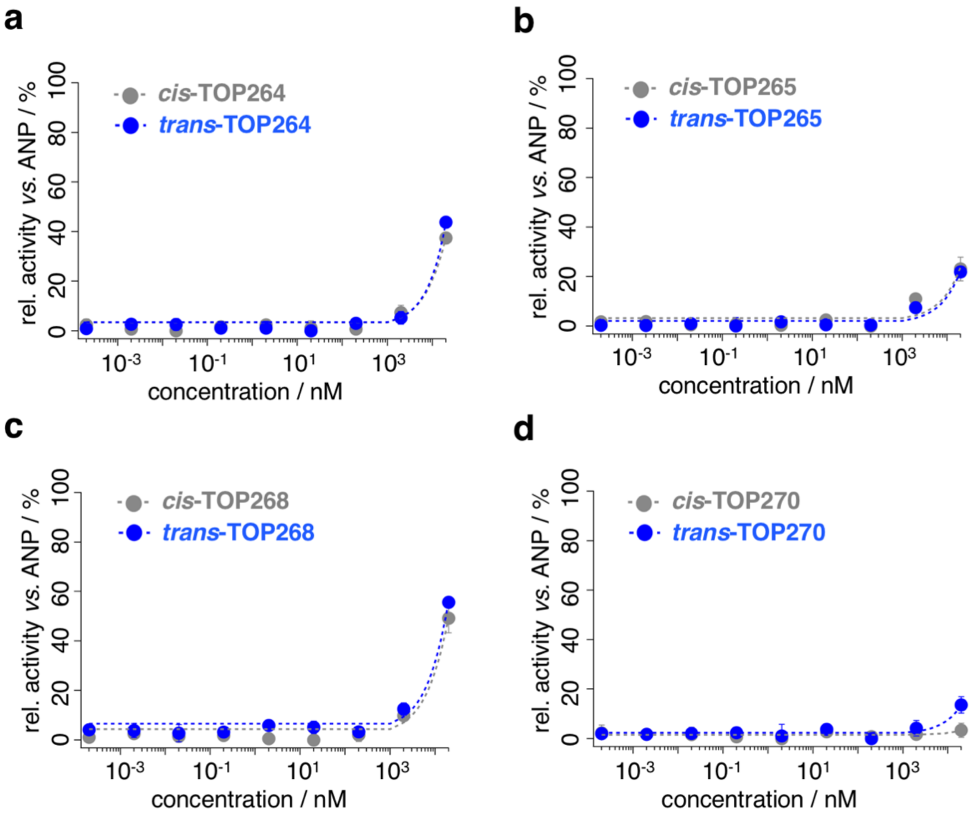


**Supplementary Figure 5:** cGMP concentration-response curves of peptides (a) TOP264, (b) -265, (c) -268 and (d) -270 recorded in YFP and NPR-A co-transfected HEK293t cells using a cisbio HTRF® kit. $EC_{50}$ values of TOP264, -265 and -268 and -270 were not calculated, nor were experiments repeated due to low activation in the μM range. cGMP dose-response curves of peptides TOP263, -266, -267 and -269 are not displayed here, due to no detectable receptor activation in the tested dose range.

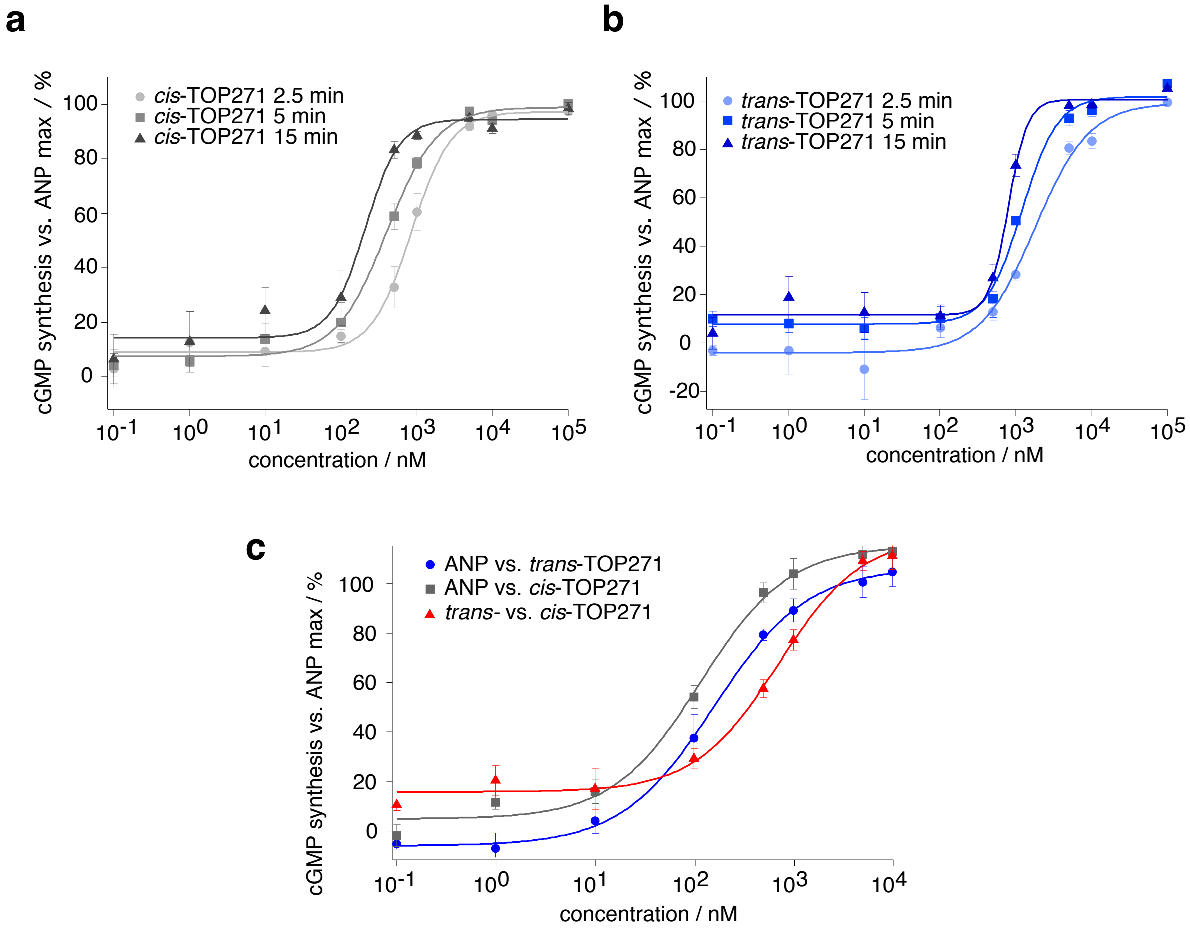


**Supplementary Figure 6:** cGMP concentration-response curves of *cis/trans*-TOP271 recorded in YFP and NPR-A co-transfected HEK293t cells using a Cisbio HTRF® kit. (a) cGMP concentration-response curves of *cis*-TOP271 at 2.5, 5 and 15 min incubation time. (b) cGMP concentration-response curves of *trans*-TOP271 at 2.5, 5 and 15 min incubation time. The $EC_{50}$ values for (a,b) are shown in Supplementary Table 3. (c) cGMP competition assays of equimolar amounts of ANP *vs. trans*-TOP271 ($EC_{50}$ = 155 ± 12 nM), ANP *vs. cis*-TOP271 ($EC_{50}$ = 113 ± 23 nM) and *trans*- *vs. cis*-TOP271 ($EC_{50}$ = 691 ± 123 nM). Values represent mean ± SEM (n = 2, 2 repeats).

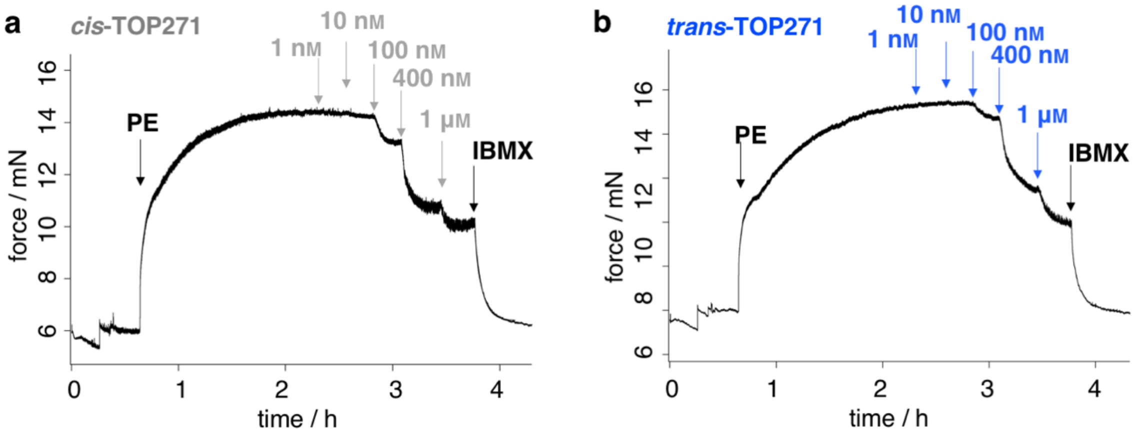


**Supplementary Figure 7:** Representative traces for aortic ring tensometry vasodilatory dose-responses recorded for *cis*- (gray, a) and *trans*-TOP271 (blue, b).

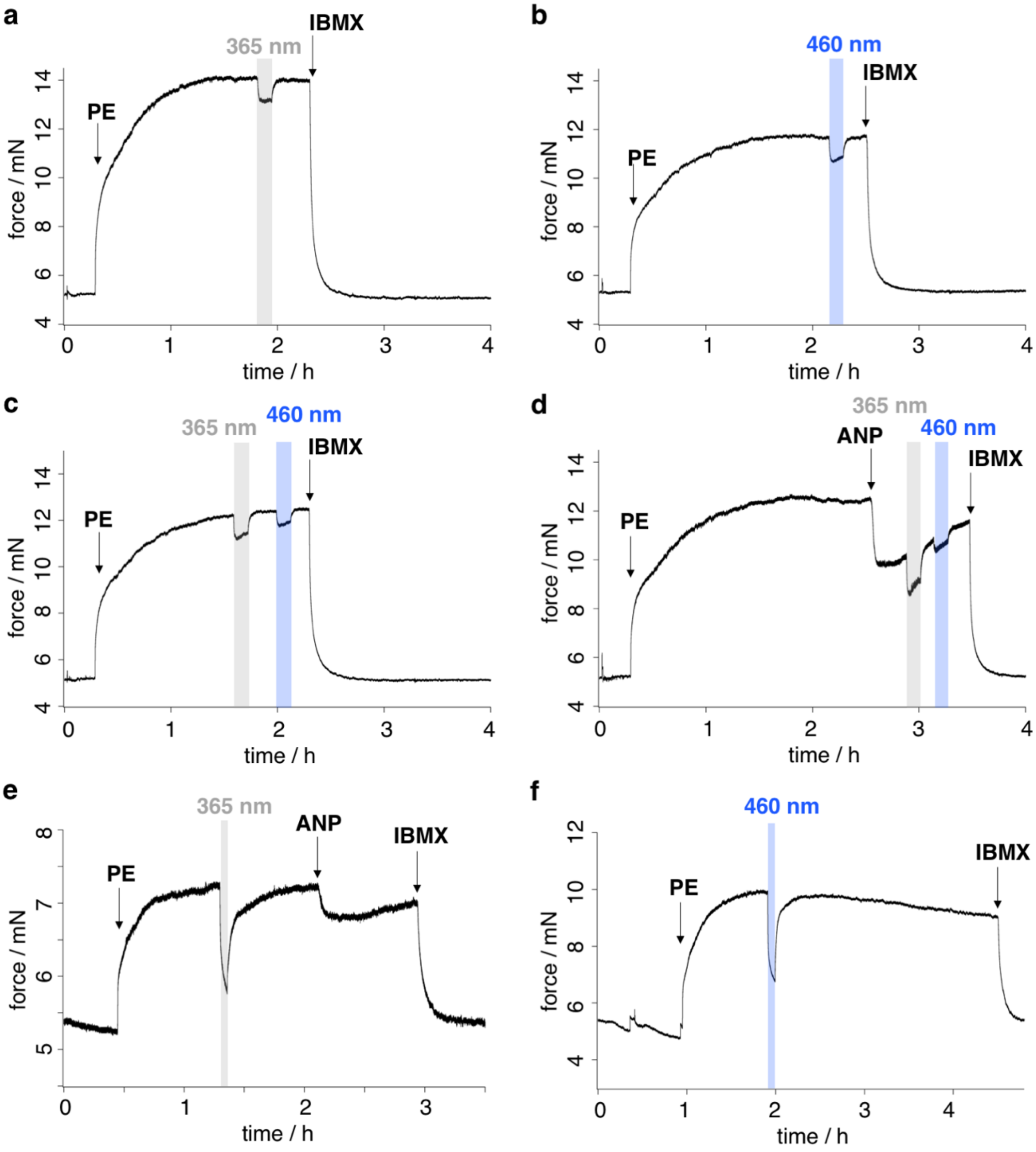


**Supplementary Figure 8:** Aortic ring tensometry illumination control experiments showing the reversibility of UV (365 nm) and blue (460 nm) light induced vasoactive responses. Rings were preconstricted with 1 μM phenylephrine (PE) and maximally dilated with 100 μM 3-isobutyl-1-methylxanthin (IBMX). To test the reversibility on dilated rings, 17 nM ANP was added in some cases. (a-d) Indirect illumination of the surrounding organ bath solution. (e,f) Direct illumination of the tissue. (a) Vasodilation with UV light (365 nm). (b) Vasodilation with blue light (460 nm). (c) Vasodilation with UV (365 nm) and blue light (460 nm). (d) Reversible dilational effect of UV (365 nm) and blue light (460 nm) on vasodilated rings (17 nM ANP). (e) Reversible effect of direct tissue illumination with UV light (365 nm). (f) Reversible effect of direct tissue illumination with blue light (460 nm).

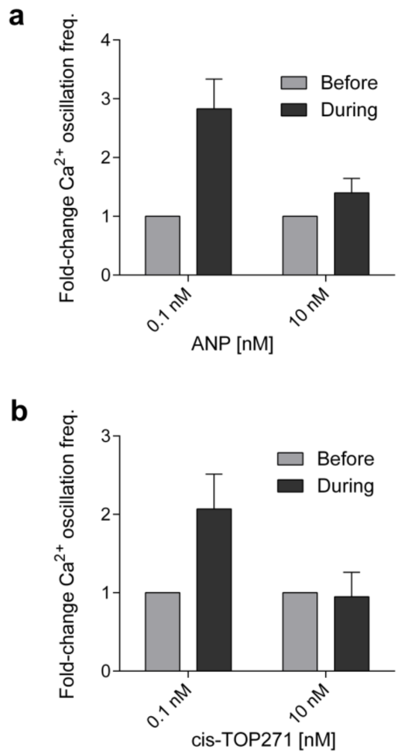


**Supplementary Figure 9:** (a) Low (0.1 nm) but not high (10 nm) concentrations of ANP stimulate increases in pancreatic beta cell $Ca^{2+}$ spiking frequency (n = 5-7 islets for each concentration). b) As for a), but *cis*-TOP271 (n = 4-7 islets for each concentration).

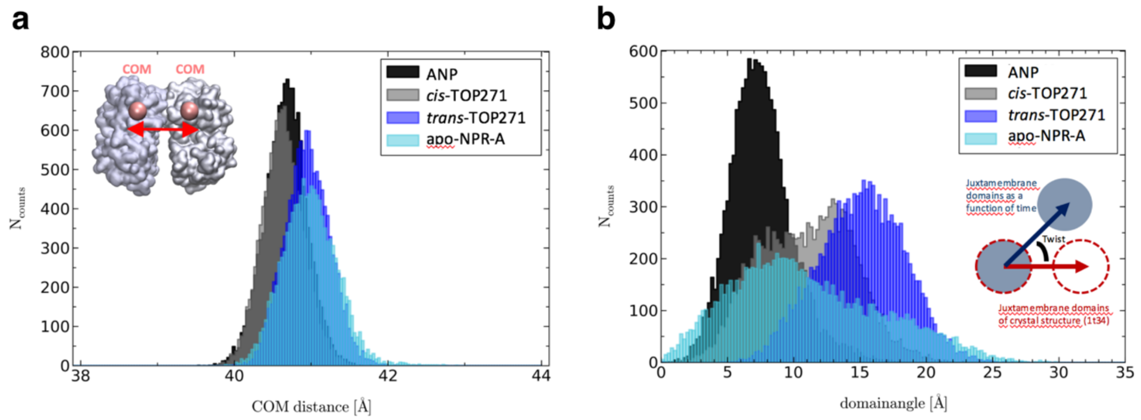


**Supplementary Figure 10:** (a) Center-of-mass distance distribution of the membrane-distal NPR-A domains A and B after 1 µs simulation show no significant ligand-dependence for bound native ANP (black), *cis/trans*-TOP271 (gray and blue, respectively) and apo-NPR-A (cyan). (b) Twist angle distributions of the membrane-proximal NPR-A subdomains with respect to the crystal structure 1t34[5] indicating only a slight isomer-dependence, with the distributions of ANP and *cis*-TOP271 (black and gray, respectively) having more overlap than ANP and *trans*-TOP271 (blue).

**Supplementary Table 1:** Photoswitching kinetics of peptides TOP263-271.

| **TOP263** | **τ (sec)** |
|---|---|
| *cis* | 67.2 ± 3.7 |
| *trans* | 46.2 ± 2.2 |
| **TOP264** | |
| *cis* | 83.6 ± 1.6 |
| *trans* | 55.1 ± 1.4 |
| **TOP265** | |
| *cis* | 71.3 ± 1.2 |
| *trans* | 50.3 ± 0.8 |
| **TOP266** | |
| *cis* | 136.6 ± 1.1 |
| *trans* | 84.8 ± 0.8 |
| **TOP267** | |
| *cis* | 84.8 ± 6.4 |
| *trans* | 62.0 ± 4.0 |
| **TOP268** | |
| *cis* | 174.3 ± 7.1 |
| *trans* | 95.7 ± 2.6 |
| **TOP269** | |
| *cis* | 167.8 ± 8.9 |
| *trans* | 98.9 ± 3.1 |
| **TOP270** | |
| *cis* | 187.0 ± 6.5 |
| *trans* | 103.2 ± 2.8 |
| **TOP271** | |
| *cis* | 163.2 ± 5.3 |
| *trans* | 95.8 ± 0.8 |

**Supplementary Table 2:** $k_{obs}$ of thermal *cis*→*trans* relaxation of peptides TOP263-271

| **Peptide** | $k_{obs}$ **× $10^{-4}$ a.u. / min** |
|---|---|
| **TOP263** | 2.825 ± 0.008 |
| **TOP264** | 0.945 ± 0.010 |
| **TOP265** | 0.316 ± 0.004 |
| **TOP266** | 2.726 ± 0.006 |
| **TOP267** | 0.942 ± 0.010 |
| **TOP268** | 2.020 ± 0.026 |
| **TOP269** | 0.275 ± 0.003 |
| **TOP270** | 1.620 ± 0.069 |
| **TOP271** | 0.975 ± 0.010 |

**Supplementary Table 3:** $EC_{50}$ values of ANP, *cis/trans*-TOP271 for cGMP synthesis at 1, 2.5, 5, 15 and 30 min.

| **Peptide** | $EC_{50,\ 1min}$ | $EC_{50,\ 2.5min}$ | $EC_{50,\ 5min}$ | $EC_{50,\ 15min}$ | $EC_{50,\ 30min}$ |
|---|---|---|---|---|---|
| **ANP** | 524 ± 216 nM | - | 28.5 ± 8.4 nM | 2.2 ± 0.4 nM | 2.0 ± 0.4 nM |
| ***cis*-TOP271** | - | 850 ± 70 nM | 403 ± 48 nM | 209 ± 47 nM | 127 ± 11 nM |
| ***trans*-TOP271** | - | 1.79 ± 0.36 uM | 1.21 ± 0.16 uM | 788 ± 61 nM | 468 ± 59 nM |

**Supplementary Table 4:** Average twist angle of NPR-A membrane-proximal domains upon ligand binding

| **Twist angle** | **Mean / °** | **Stddev / °** |
|---|---|---|
| **ANP** | 7.56 | 2.37 |
| ***cis*-TOP271** | 10.99 | 3.51 |
| ***trans*-TOP271** | 15.10 | 3.25 |
| **apo-NPR-A** | 11.07 | 5.58 |

**Supplementary Table 5:** $^{1}H/^{13}C$-NMR chemical shifts of *trans*-TOP271

| **Residue** | **$^{1}$H chem. shifts [ppm]** | **$^{13}$C chem. shifts [ppm]** |
|---|---|---|
| **Ser1** | Hα 4.09, Hβ* 3.95 | Cα 56.8, Cβ 62.6 |
| **Leu2** | NH 8.52, Hα 4.35, Hβ* 1.55, Hγ 1.54, Hδa 0.82, Hδb 0.86 | Cα 55.0, Cβ 42.1, Cγ 26.4, Cδa 23.0, Cδb 23.9 |
| **Arg3** | NH 8.06, Hα 4.26, Hβa 1.79, Hβb 1.69, Hγa 1.54, Hγb 1.55, Hδ* 3.07, Hε 7.07 | Cα 55.6, Cβ 30.7, Cγ 26.7, Cδ 42.8 |
| **Arg4** | - | - |
| **Ser5** | NH 7.96, Hα 4.29, Hβa 3.62, Hβb 3.69 | Cα 58.1, Cβ 63.3 |
| **Ser6** | NH 7.90, Hα 4.22, Hβa 3.64, Hβb 3.59 | Cα 58.4, Cβ 63.1 |
| **Cys7** | NH 8.52, Hα 4.59, Hβ* 2.72 | Cα 53.4, Cβ 38.0 |
| **Phe8** | NH 8.11, Hα 4.56, Hβa 3.11, Hβb 2.96, Hδ* 7.25, Hε* 7.18, Hζ 7.11 | Cα 57.6, Cβ 38.9, Cδ* 130.8, Cε* 131.2, Cζ 129.2 |
| **Gly9** | NH 8.18, Hαa 3.73, Hαb 3.71 | Cα 44.8 |
| **Gly10** | NH 8.14, Hαa 3.82, Hαb 3.81 | Cα 44.9 |
| **Arg11** | NH 7.66, Hα 4.18, Hβa 1.60, Hβb 1.49, Hγ* 1.35, Hδ* 3.00, Hε 6.99 | Cα 55.2, Cβ 30.5, Cγ 26.7, Cδ 42.7 |
| **Met12** | NH 7.57, Hα 4.44, Hβa 2.00, Hβb 1.91, Hγa 2.47, Hγb 2.39, Hε* 1.98 | Cα 54.3, Cβ 31.9, Cγ 31.5, Cε 16.0 |
| **Asp13** | NH 7.76, Hα 4.47, Hβa 2.97, Hβb 2.86 | Cα 57.3, Cβ 38.8 |
| **Arg14** | NH 7.76, Hα 4.20, Hβa 1.63, Hβb 1.52, Hγ* 1.38, Hδ* 3.02, Hε 7.01 | Cα 55.2, Cβ 30.6, Cγ 26.3, Cδ 42.7 |
| **Ile15** | NH 7.82, Hα 3.99, Hβ 1.76, Hγ1a 1.04, Hγ1b 1.02, Hγ2* 0.75, Hδ1* 0.72 | Cα 61.6, Cβ 37.8, Cγ1 27.0, Cγ2 16.5, Cδ1 11.8 |
| **Gly16** | NH 8.09, Hα* 3.87 | Cα 44.8 |
| **Ala17** | NH 7.87, Hα 4.23, Hβ* 1.31 | Cα 52.4, Cβ 18.5 |
| **Gln18** | NH 8.15, Hα 4.31, Hβa 2.12, Hβb 1.98, Hγ* 2.30 | Cα 55.7, Cβ 29.1, Cγ1 33.5 |
| **AMPP** | NH 8.19, H2* 4.41, H4 7.66, H6 7.68, H7 7.46, H8 7.38, H12 7.73, H14 7.42, H15 7.49, H16 7.71, H17a 3.80, H17b 3.73 | C2 44.9, C4 123.1, C6 123.8, C7 131.9, C8 132.5, C12 125.1, C14 134.6, C15 131.9, C16 123.8, C17 43.9 |
| **Cys23** | NH 8.52, Hα 4.64, Hβ* 2.69 | Cα 53.2, Cβ 38.1 |
| **Asn24** | NH 7.88, Hα 4.51, Hβa 2.98, Hβa 2.91 | Cα 57.3, Cβ 39.0 |
| **Ser25** | NH 8.24, Hα 4.42, Hβa 3.88, Hβa 3.74 | Cα 58.1, Cβ 63.5 |
| **Phe26** | NH 7.62, Hα 4.46, Hβa 3.02, Hβa 2.83, Hδ* 7.13, Hε* 7.04, Hζ 7.20 | Cα 56.7, Cβ 38.1, Cδ* 130.7, Cε* 131.2, Cζ 129.3 |
| **Arg27** | - | - |
| **Tyr28** | NH 7.63, Hα 4.46, Hβa 3.02, Hβa 2.84, Hδ* 7.01, Hε* 6.73, Hζ 7.20 | Cα 58.1, Cβ 38.1, Cδ* 132.7, Cε* 117.5 |

## *4. References*


1. A. Aemissegger, V. Kräutler, W. F. van Gunsteren, and D. Hilvert, *J. Am. Chem. Soc.*, 2006, **127**, 2929–2936.
2. T. Podewin, M. Rampp, I. Turkanovic, K. Karaghiosoff, W. Zinth, and A. Hoffmann-Röder, *Chem. Commun.*, 2015, **51**, 4001–4004.
3. J. Broichhagen, T. Podewin, H. Meyer-Berg, Y. von Ohlen, N. R. Johnston, B. J. Jones, S. R. Bloom, G. A. Rutter, A. Hoffmann-Röder, D. J. Hodson, and D. Trauner, *Angew. Chem. Int. Ed.*, 2015, **54**, 15565–15569.
4. Y.-C. Chao, C.-J. Cheng, H.-T. Hsieh, C.-C. Lin, C.-C. Chen, and R.-B. Yang, *Biochem. J.*, 2010, **432**, 267–273.
5. H. Ogawa, Y. Qiu, C. M. Ogata, and K. S. Misono, *J. Biol. Chem.*, 2004, **279**, 28625–28631.
6. J. Wang, W. Wang, P. A. Kollman, and D. A. Case, *J. Mol. Graph. Model.*, 2006, **25**, 247–260.
7. J. Wang, R. M. Wolf, J. W. Caldwell, P. A. Kollman, and D. A. Case, *J. Comput. Chem.*, 2004, **25**, 1157–1174.
8. A. D. Case et al., *University of California, San Francisco*, 2014.
9. W. Jorgensen and J. Chandrasekhar, *J. Chem. Phys.*, 1983, **79**, 926–935.
10. U. Essmann, L. Perera, and M. Berkowitz, *J. Chem. Phys.*, 1995, **103**, 8577–8593.
11. H. Berendsen and J. Postma, *J. Chem. Phys.*, 1984, **81**, 3684–3690.
12. S Miyamoto and PA Kollman, *J. Comp. Chem.*, 1992, **13**, 952–962.
13. C. Hopkins, L. S. Grand, and R. Walker, *J. Chem. Theory Comput.*, 2015, **11**, 1864–1874.
14. D. R. Roe and T. E. Cheatham, *J. Chem. Theory Comput.*, 2013, **9**, 3084–95.
15. W. Humphrey, A. Dalke, and K. Schulten, *J. Mol. Graph.*, 1996, **14**, 33–38.
16. D. Groneberg, P. König, A. Wirth, and S. Offermanns, *Circulation*, 2010, **121**, 401–409.
